# Efficacy of Herbal Medicines Intervention for Colorectal Cancer Patients With Chemotherapy-Induced Gastrointestinal Toxicity — a Systematic Review and Meta-Analysis

**DOI:** 10.3389/fonc.2021.629132

**Published:** 2021-03-25

**Authors:** Yuanyuan Chen, Chien-shan Cheng, Hor-Yue Tan, Chi Wing Tam, Ning Wang, Yibin Feng

**Affiliations:** Li Ka Shing Faculty of Medicine, School of Chinese Medicine, The University of Hong Kong, Hong Kong, China

**Keywords:** colorecal cancer, herbal medicine, traditional medicine, gastrointestinal toxicity, chemotherapy induced nausea and vomiting, chemotherapy induced diarrhea, chemotherapy induced gastrointestinal toxicity, chemotherapy induced anorexia

## Abstract

**Purpose:** Chemotherapy-induced gastrointestinal (CIGI) toxicity affects the quality of life of patients with colorectal cancer (CRC) and the clinical application of treatment drugs. This review aims to evaluate the efficacy of traditional herbal medicines (HMs) in alleviating symptoms of CIGI toxicity (including nausea and vomiting, anorexia, diarrhea, constipation, oral mucositis, abdominal pain, and abdominal distension), and to explore further individual herb or herbal combinations in alleviating the CIGI toxicity.

**Methods:** Nine electronic databases were screened from 2010 to 2020. Twenty-two randomized controlled trials with a total of 1,995 patients evaluating the complementary efficacy of HMs with chemotherapy compared with chemotherapy-alone were included. Further, sensitivity analyses of orally administered multi-ingredient HM interventions were explored based on the composition of HM interventions.

**Results:** The meta-analysis showed that HM treatment combined with chemotherapy significantly alleviated the overall CIGI toxicity (RR = 0.78 [0.72, 0.84], *p* < 0.001, *I*^2^ = 44%), nausea and vomiting (RR = 0.74 [0.66, 0.82], *p* < 0.001, *I*^2^ = 35%), diarrhea (*P* = 0.02, RR = 0.64, 95% CI = 0.44–0.93, *I*^2^ = 50%), oral mucositis (RR = 0.65 [0.48, 0.88], *P* = 0.005, *I*^2^ = 24%), and abdominal distension (RR = 0.36 [0.18, 0.73], *P* = 0.004, *I*^2^ = 0%). However, no statistically significant effects of HMs were shown in studies with a double-blind design for CIGI toxicity. Based on the ingredients of the HMs, further sensitivity analyses identified five herbs [*Glycyrrhiza uralensis* Fisch., *Atractylodes macrocephala* Koidz., *Astragalus membranaceus* (Fisch.) Bge., *Codonopsis pilosula* (Franch.) Nannf., and the pericarp of *Citrus reticulata* Blanco.] that were associated with significant reductions in CIGI toxicity.

**Conclusion:** A statistically significant effect of HMs combined with chemotherapy on alleviating the overall CIGI toxicity, nausea and vomiting, diarrhea, oral mucositis, or abdominal distension is only shown in studies without a double-blind design. Further well-designed, double-blinded, large-scaled randomized controlled trials (RCTs) are warranted to comprehensively evaluate the treatment efficacy. Further clinical research that includes the five herbs with chemotherapy for patients, the safety of the combinations of these herbs, and the potential synergistic effects of these combinations of herbs should be conducted.

## Introduction

Colorectal cancer (CRC) is considered the second most frequently diagnosed carcinoma in women and the third in men worldwide. There are 1.8 million patients newly diagnosed with CRC in 2018 (1). Chemotherapy 5-fluorouracil (FU) has been the backbone of treatment in patients with CRC for more than half a century (2), and the combination of 5-FU with irinotecan and oxaliplatin has become the standard of therapy for patients with metastatic CRC (mCRC) in the late 1990's (3). However, up to 80% of patients with CRC receiving 5-FU based adjuvant therapy develop gastrointestinal (GI) toxicity, which is currently without a widely effective treatment strategy (4, 5). Common symptoms in CIGI include nausea and vomiting, diarrhea, abdominal pain, abdominal bleeding, ulcerative lesions along the GI tract, etc. (6, 7). Some GI symptoms, such as nausea and vomiting caused by chemotherapy, are well-managed by using potent anti-emetic drugs (8). However, other common GI symptoms reported by patients with cancer such as altered taste, anorexia, dysphagia, reflux, regurgitation, borborygmi, bloating, constipation, diarrhea, tenesmus, mucus discharge, steatorrhea, weight loss, etc., are still lacking optimal management (7, 8). These symptoms often lead to a reduction of therapeutic dose, compromised clinical efficacy of treatment drugs, and impinge on the quality of life of patients. Severe complications of CIGI toxicity such as bacteremia and sepsis interfere with chemotherapy prompting dose reduction and, in profound cases, cessation of therapy.

In order to maintain the tolerance of chemotherapy while preserving the quality of life of patients, the hunt for a complementary treatment for the alleviation of CIGI toxicity becomes crucial. Chinese traditional herbal medicine (HM), which refers to the utilization of plants or plant-derived materials, represents one of the most commonly used complementary treatments of GI toxicity. A large number of herbal formulae such as Si-Jun-Zi decoction, Ping-Wei-San, Shen-Ling-Bai-Zhu-San, etc., have been used in China for over 1,800 years for treating GI toxicity. The use of HM is based on the sophisticated theory of TCM and has undergone long-term repeated confirmation (9). Recent studies on cell line, animal, and clinical trials have shown that some HMs are potentially effective in alleviating CIGI toxicity. Previous systematic reviews and meta-analyses have reported that the combination of HMs with chemotherapy, could alleviate CIGI toxicity in CRC (10–13). However, these systematic reviews and meta-analyses mainly focused on single symptoms such as nausea and/or vomiting (10–13), diarrhea (10–13), and anorexia (11). An overall assessment of the CIGI toxicity of HM treatments is necessary since multiple symptoms of CIGI toxicity may occur in individual patients, and usually, one single HM prescription with combinations is used. Besides, studies that assess the individual symptoms usually focus more on the efficacy of HMs across different types of cancers, which may cause high heterogeneity due to the different severity of GI symptoms. In addition, most of the studies that assess the efficacy of HM treatment do not adopt a double-blind design. This may introduce performance bias into a meta-analysis, and thus overestimate the efficacy of HMs in alleviating individual symptoms.

The review aims to assess the efficacy of HMs in alleviating the symptoms of CIGI toxicity by evaluating the complementary efficacy of HMs with chemotherapy compared with chemotherapy alone. An overall analysis of CIGI toxicity and sub-analysis on the following symptoms: nausea and vomiting, diarrhea, anorexia, constipation, oral mucositis, abdominal pain, and abdominal distension will be conducted. In addition, this review will stratify data analysis for CIGI toxicity with a double-blind design and studies without a double-blind design. Further sensitivity analysis of orally administered multi-ingredient HM interventions will be conducted to explore individual herb or herbal combinations in alleviating the CIGI toxicity.

## Methods

This systematic review was performed in accordance with the Preferred Reporting Items for Systematic Reviews and Meta-Analysis (PRISMA). This study protocol was registered in the International Prospective Register of Systematic Reviews (PROSPERO) with registration number CRD42020201981.

### Data Sources and Searches

Nine electronic databases, including PubMed, Cochrane Library, EMBASE, ISI Web of Science, Comprehensive Journal Index and Additional Resources for Nursing and Allied Health Professionals (CINAHL Plus), AMED, WanFang Data, and China National Knowledge Infrastructure (CNKI) were searched for CIGI toxicity in patients with CRC. There were no restrictions on language. Only randomized controlled trials (RCTs) evaluated the effects of the combination of the HMs with chemotherapy in comparison with the same chemotherapy regimen were included. The complete search strategy used for the bibliographic databases is provided in Appendix 1.

### Eligibility Criteria

Randomized controlled studies with two or more arms studies were included according to the following criteria: (1) studies examined adults who had been diagnosed with CRC by pathologists; (2) studies assessed at least one of the symptoms of CIGI toxicity, including nausea and vomiting, diarrhea, anorexia, constipation, oral mucositis, abdominal pain, and abdominal distension; (3) studies that used HM, including a single substance or multi-ingredient formulation as an interventional group without administration restriction; (4) studies that used chemotherapy such as folinic acid, fluorouracil and irinotecan (FOLFIRI), folinic acid, fluorouracil and oxaliplatin (FOLFOX), and other regimens combined with HMs in the intervention group, and compared with the same chemotherapy in the control group. Anti-emetic drugs were allowed for use; and (5) studies that measured CIGI toxicity were assessed by toxicity criteria recommended by WHO (14), the National Cancer Institute Common Terminology Criteria for Adverse Events (15), the Guidelines for Clinical Research of New Chinese Medicines (16), or any validated criteria. GI toxicity was either a primary or a secondary outcome of the study.

### Study Identification and Data Extraction

Two independent reviewers extracted the following information from each study: first author and year of publication; sample size; intervention and control; treatment dosage and duration; and toxicity assessment. Discrepancies were resolved through consensus discussion and the suggestion of a third reviewer.

### Risk of Bias and Quality Assessment

The RoB of each included trial was evaluated with the Cochrane Collaboration's tool for assessing the RoB in randomized trials. RoB was assessed by two reviewers. The following domains were assessed: random sequence generation, allocation concealment, blinding of participants and personnel, blinding of outcome assessment, completeness of data sets, selective outcome reporting, and other bias. Each of the domains was judged “low RoB,” “high RoB,” and “unclear RoB.”

Each study was also assessed using the Jadad scale for assessing bias (17). This scale contained five questions; (i) randomization, (ii) appropriate method for randomization, (iii) double-blinding, (iv) appropriate method for double-blinding, and (v) description of dropouts and withdrawals. A score of 0 or 1 was given to each question, with higher scores representing higher methodological quality. Studies with a score of ≥ 3 were considered high-quality clinical trials, and studies with a score of < 3 were considered low-quality (18).

### Statistical Analysis

Review Manager (RevMan) 5.1 was used to conduct the meta-analysis. Review methods were based on Cochrane Handbook 6.1 (19). Data were indicated as risk ratio (RR) with 95% confidence interval (95% CI) or mean difference (MD) in a fixed-effects model or a random-effect model. Heterogeneity was measured using *I*^2^. A random-effect model was used if *I*^2^ is not < 50%. Studies that measured CIGI toxicity assessed by RR were subjected to the meta-analysis. Studies assessed by MD were analyzed separately. Funnel plots were generated to investigate publication bias (19).

### Subgroup Analyses

To assess the efficacy of HMs for alleviating CIGI toxicity induced by chemotherapy in patients with CRC, subgroup and sensitivity analyses were explored for each symptom of CIGI toxicity: nausea and vomiting, diarrhea, anorexia, constipation, oral mucositis, abdominal pain, and abdominal distension. Data analysis for each symptom was stratified by studies with a double-blind design and studies without a double-blind design.

### Sensitivity Analyses of Orally Administered HM Interventions

In order to explore individual herb or herbal combinations in alleviating the CIGI toxicity, further sensitivity analyses of orally administered multi-ingredient HM interventions were explored based on the composition of HM interventions. Chen et al. reasoned that the pooled RR outcomes of multiple studies that employed the same herb or the same herbal combination reflected whether a particular herb or combination in the intervention was effective or not (20). In our review, the method described by Chen et al. was used to explore individual herb or herbal combinations in alleviating the CIGI toxicity. Therefore, herbs or herbal combinations that had RR results smaller than the pooled RR were identified to show potential for further research into interventions.

The approach was described as following: at Level 1, single herbs present in more than one study were identified. Studies that contained the same herbs were considered a subgroup, and the pooled RR (with 95% CI and *I*^2^) was calculated for each subgroup of studies. Only subgroups that had significant pooled RRs with *I*^2^ < 30% were considered for higher-level combinations. At Level 2, single herbs with significant pooled RRs and *I*^2^ < 30% were paired up, and the RRs of pairs were calculated for each pool. Significant results with *I*^2^ < 30% were noted. At Level 3 and above, combinations of 3, and more herbs were generated. The RRs of combinations were calculated for each pool, and significant results with *I*^2^ < 30% were noted. The pooled RRs at each level were indicated in ascending order. Besides, only advanced combinations of herbs were included. For example, in the review, all the HM interventions that contained *G. uralensis* + *C. lacryma* also included *C. pilosula*, so no contribution to the RR from *G. uralensis* + *C. lacryma* was shown at Level 2.

According to Chen et al., the following selection criteria were used in order to select herbs for further research (20): (1) the RR result of the group of more than one study that included the same herb(s) in the HM interventions was significant with *I*^2^ < 30%; (2) the RR was equal or lesser than the total pooled RR for the multi-ingredient HM interventions; and (3) the herb was contained at multiple levels of combination with consistently significant RR results, or the herbal combination had RRs that were lower than those of the herbs individually.

## Results

### Study Selection

**Figure 1:**
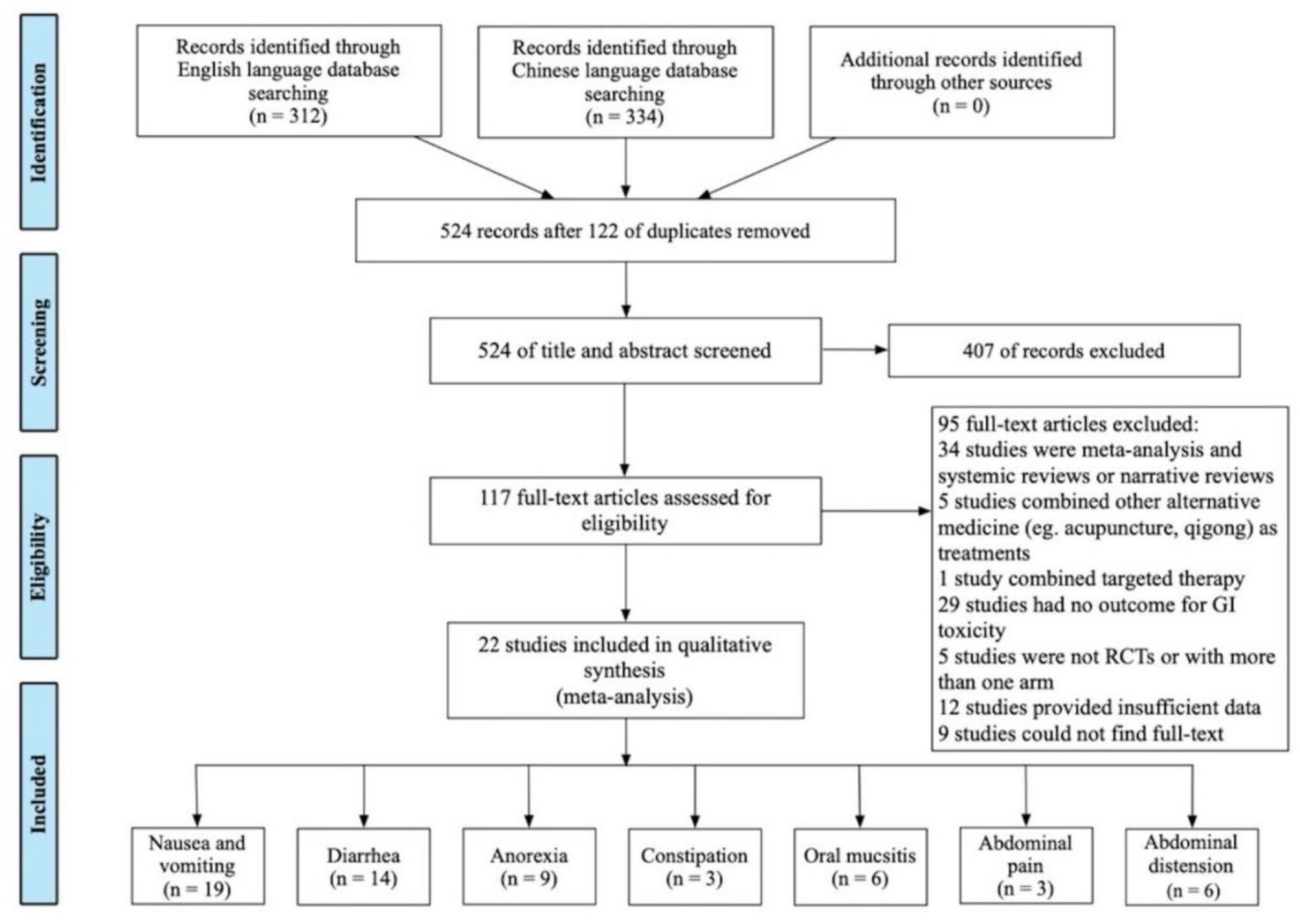
Study selection flowchart based on PRISMA.

The literature search retrieved 646 records; 122 of them were duplicates (Figure 1). After identifying the unduplicated studies, 117 studies were evaluated, and 94 studies were further excluded. Finally, 22 studies involving a total of 1,995 patients were recruited and included in the qualitative synthesis. Included studies were published from 2010 to 2020, and most of them were conducted in Mainland China. Nineteen studies reported nausea and vomiting (21–39); 14 studies reported diarrhea (21, 24, 25, 27, 29, 30, 32–34, 36–40); 9 studies reported anorexia (26, 27, 31–33, 35, 36, 38, 39); 3 studies reported constipation (21, 36, 39); 6 studies reported oral mucositis (21, 24, 27, 29, 39, 41); 3 studies reported abdominal pain (33, 39, 40); and 6 studies reported abdominal distension (26, 32, 33, 39, 40, 42). Study characteristics are presented in Table 1.

**Table 1:** Characteristics of randomized controlled trials of HMs combined with chemotherapy for colorectal cancer with GI toxicity incidence as an outcome.

**References**	**GI symptom(s)**	**Sample size**	**Chemotherapy regimen; dose; anti-emetic drug**	**HM intervention; dosage and duration**	**Control group**	**Toxicity assessment**	**Jadad scores**
Xu et al. (21)	Nausea and vomiting, diarrhea, constipation, oral mucositis	100	FOLFOX4 regimen: iv LV, 200 mg/m^2^, days 1–2; iv bolus 5-FU, 400 mg/m^2^, days 1–2; continuous iv 5-FU, 600 mg/m^2^, days 1–2; iv Ox. 85 mg/m^2^ repeated every 2 weeks; granisetron. Treatment was biweekly administered.	Aidi Injection 60–80 ml, iv, once daily, plus FOLFOX4	FOLFOX-4 regimen	WHO criteria	1
Liu (40)	Diarrhea, abdominal pain, abdominal distension	76	XELOX regimen: oral Xeloda, 2,500 mg/m^2^, 2 weeks; iv Ox. 85 mg/m^2^, 2 weeks.	HM (decoction) twice daily plus XELOX	XELOX regimen	NS	3
Ji (22)	Nausea and vomiting	86	FOLFOX4: iv Ox. 85 mg/m^2^, 2 h, day 1; iv LV, 200 mg/m^2^, 2 h, days 1–2; iv 5-FU, 400 mg/m^2^, days 1–2; continuous iv 5-FU, 22 h, 600 mg/m^2^. Treatment was biweekly administered.	Fuzhengxiaoji (decoction) twice daily, plus FOLFOX4 regimen	FOLFOX4 regimen	NS	3
Li et al. (23)	Nausea and vomiting	72	FOLFOX4: iv Ox. 85 mg/m^2^, 2 h, day 1; iv LV, 200 mg/ m^2^, 2 h, days 1–2; iv 5-FU, 400 mg/m^2^, days 1–2; continuous iv 5-FU, 22 h, 600 mg/m^2^. Treatment was biweekly administered.	Fuzhengxiaoji (decoction) twice daily, plus FOLFOX4 regimen	FOLFOX4 regimen	NS	3
Hu (24)	Vomiting, diarrhea, oral mucositis	72	IFL regimen: iv irinotecan 180 mg/m^2^ + saline 250 ml, 2 h, day 1; iv LV, 300 mg/m^2^, days 2–5; iv 5-FU, 600 mg/m^2^, day 2–5.	Shenlinbaozhusan (decoction) twice daily, plus IFL regimen	IFL regimen	WHO criteria	3
Hu (42)	Abdominal distension	72	NS	Jianpizhuyu (decoction) twice daily, plus chemotherapy	Chemotherapy	Recommendations of the ESICM Working Group on Abdominal Problems	3
Xing and Wang (25)	Nausea and vomiting, diarrhea	70	XELOX regimen: iv Ox. 130 mg/m^2^ + 5% glucose injection 500 ml, 2 h, day 1; oral Xeloda, 1,800 mg/m^2^, days 1–14. Treatment was repeated every 3 weeks.	Jianpizhuyu (decoction) twice daily, plus XELOX regimen	XELOX regimen	Guidelines for clinical research of new Chinese medicines	3
Huang and Xu (26)	Nausea and vomiting, anorexia, abdominal distension	46	FOLFOX regimen: iv Ox. 130 mg/m^2^, day 1; iv LV, 200 mg/ m^2^, days 1–5; iv 5-FU, 300 mg/m^2^, days 1–5. Treatment was repeated every 2 weeks.	Jianpizhuyu (decoction) twice daily, plus FOLFOX regimen	FOLFOX regimen	Guidelines for clinical research of new Chinese medicines	3
Chen and Shen (27)	Vomiting, diarrhea, anorexia, oral mucositis	120	FOLFOX4 regimen: L-OHP 85 mg/m^2^, 2 h, day 1; iv LV, 200 mg/m^2^, 2 h, days 1–2; iv 5-FU, 400 mg/m^2^, days 1–2; continuous iv 5-FU, 22 h, days 1–2, 600 mg/m^2^. Treatment was repeated every 2 weeks. XELOX regimen: L-OHP 85 mg/m^2^, 2 h, day 1; oral CapeOX, 850–1,000 mg/m^2^, days 1–14. Treatment was repeated every 21 days.	Qisheng mixture, 150 ml, twice daily, plus FOLFOX4 regimen or XELOX regimen	FOLFOX4 regimen or XELOX regimen	Guidelines for clinical research of new Chinese medicines	3
Xiao and Yang (28)	Nausea and vomiting		FOLFOX4 regimen	Jiaweisijunzi (decoction) twice daily, plus FOLFOX4 regimen	FOLFOX4 regimen	WHO criteria	3
Zhang et al. (29)	Nausea and vomiting, diarrhea, oral mucositis	120	XELOX regimen: iv Ox. 135 mg/m^2^, 3 h, day 1; oral Xeloda, 1,000 mg/m^2^, days 1–14. Treatment was repeated every 3 weeks.	Xihuang capsules twice daily, plus XELOX regimen	XELOX regimen	WHO criteria	3
Zhao et al. (41)	Oral mucositis	80	FOLFOX regimen: L-OHP 130 mg/m^2^ +5% glucose injection 250 ml, 2 h, day 1; iv LV, 100 mg/m^2^, 2 h, days 1–5; iv 5-FU, 100 mg/m^2^,days 1–5.	HM (decoction) twice daily, plus FOLFOX regimen	FOLFOX regimen	WHO criteria	3
Nan and Li (30)	Nausea and vomiting, diarrhea	48	iv Irinotecan HCl, 160 mg/m^2^, 90 min, day 1; iv Raltitrexed, 3 mg/m^2^, day 2	Shenlinbaizhusan (decoction) twice daily, plus Irinotecan + Ramucirumab	Irinotecan + Ramucirumab	Guidelines for clinical research of new Chinese medicines	3
Song et al. (31)	Nausea and vomiting, anorexia	43	FOLFOX regimen: iv Ox. 85 mg/m^2^, 2 h, days 1–5; iv LV, 400 mg/m^2^, 2 h, days 1–5; iv 5-FU, 1 g, days 1–5. Treatment was repeated every 3 weeks.	HM twice daily, plus FOLFOX regimen	FOLFOX regimen	NS	3
Zhang and Han (32)	Nausea and vomiting, diarrhea, anorexia, abdominal distension	60	mFOLFOX6 regimen: iv Ox. 85 mg/m^2^, 2 h, day 1; iv LV, 400 mg/m^2^, 2 h, day 1; iv 5-FU, 400 mg/m^2^, day 1; continuous iv 5-FU, 2,400 mg/m^2^, 46 h, days 1–2. Treatment was repeated every 2 weeks.	Yiqisanjie Fang decoction twice daily, plus mFOLFOX6 regimen	mFOLFOX6 regimen	Guidelines for clinical research of new Chinese medicines	3
Peng et al. (33)	Nausea and vomiting, diarrhea, anorexia, abdominal pain, abdominal distension	40	iv Ox. 100 mg/m^2^ + 5% glucose injection 250 ml, 2 h, day 1; iv LV, 200 mg/m^2^ + 0.9% sodium chloride solution 250 ml, days 1–5; iv 5-FU, 750 mg/m^2^ + 5% glucose injection 500 ml, 6 h, days 1–5. Treatment was repeated every 21 days.	HM (decoction) twice daily, plus FOLFOX regimen	FOLFOX regimen	WHO criteria	3
Zeng (34)	Nausea and vomiting, diarrhea	60	FOLFOX4 regimen: iv LV, 200 mg/m^2^, days 1–2; iv bolus 5-FU, 400 mg/m^2^, days 1–2; continuous iv 5-FU, 600 mg/m^2^, 22h, days 1–2; iv Ox. 85 mg/m^2^, day 1. Treatment was repeated every 2 weeks.	HM (decoction) twice daily, plus FOLFOX4 regimen	FOLFOX4 regimen	NS	3
Matsuda et al. (39)	Nausea, vomiting, diarrhea, anorexia, constipation, oral mucositis, abdominal pain	90	FOLFOX, FOLFIRI, or XELOX regimen	Hangeshashinto (TJ-14) (powder) 2.5 g × 3 times per day for a total daily dose of 7.5 g, plus chemotherapy	Chemotherapy plus placebo	WHO criteria	5
Motoo et al. (35)	Nausea and vomiting, anorexia	52	CapeOX regimen: capecitabine, 2,400 mg/m^2^; oxaliplatin, 130 mg/m^2^, at 3 week intervals.	Ninjin'yoeito (NYT) (powder) 9 g per day in divided doses 2 or 3 times a day, plus CapeOX regimen	CapeOX regimen	National Cancer Institute Common Terminology Criteria for Adverse Events (NCI-CTCAE v4.0)	3
Kono et al. (36)	Nausea, vomiting, diarrhea, anorexia, constipation	89	FOlFOX4 regimen: iv LV, 100 mg/m^2^, 2 h; 5-FU as a bolus, 400 mg/m^2^, 22 h infusion of 5-FU, 600 mg/m^2^, days 1–2, iv oxaliplatin, 85 mg/m^2^, 2 h, day 1. Treatment was repeated every 2 weeks. mFOlFOX6 regimen: iv LV, 200 mg/m^2^, 2 h; 5-FU as a bolus 400 mg/m^2^, 46 h infusion of 5-FU, 2,400 mg/m^2^; iv oxaliplatin, 85 mg/m^2^, 2 h, day 1. Treatment was repeated every 2 weeks.	Goshajinkigan (TJ-107) (powder) 2.5 g × 3 times per day for a total daily dose of 7.5 g, plus chemotherapy	Chemotherapy plus placebo	NS	5
Liu et al. (37)	Nausea, vomiting, diarrhea	120	FOLFOX4 regimen: iv Ox. 85 mg/m^2^, day 1, 3 h; 250 mL dextrose 5%, eucovorin, 200 mg/m^2^, 2 h; Fluorouracil, 400 mg/m^2^, 24 h; fluorouracil 2,400 mg/m^2^/d, days 1–2.	Guilongtongluofang (decoction) once daily, plus chemotherapy	Chemotherapy plus placebo	National Cancer Institute's (NCI) common toxicity criteria (CTC)	5
Oki et al. (38)	Nausea, vomiting, diarrhea, anorexia	186	mFOLFOX6 regimen	Goshajinkigan (powder) 2.5 g × 3 times per day for a total daily dose of 7.5 g, plus mFOLFOX6 regimen	mFOLFOX6 regimen plus placebo	National Cancer institute common terminology criteria for adverse events (NCI-CTCAE)	5

*NS, not stated; h, hours; ID, intravenous drip; HM, Herbal medicine; 5-FU, 5-Fluorouracil; LV, Leucovorin; Ox., Oxaliplatin; FOLFOX, Ox. + 5-FU + LV; XELOX, Ox. + Capecitabine; CapeOX, Capecitabine + Ox; IFL, Irinotecan + LV + fluorouracil; L-OHP, Oxaliplatin; WHO, World Health Organization; mFOLFOX, modified FOLFOX; ESICM, European Society of Intensive Care Medicine*.

### Study Characteristics

The following study characteristics are recorded in Table 1: CIGI symptoms, sample sizes, interventions, doses, schedules, controls, outcome measures, and Jaded scores. Of the 22 eligible studies, 18 studies originated from China and 4 from Japan. The mean age of included participants was between 44 and 69 years. Most of them had stage III or stage IV cancer. Formulations of HMs used in the 22 studies included decoctions, injection, capsules, powder, and mixture. Among them, one study (21) used injection, one study (29) used capsules, four studies (35, 36, 38, 39) used power, and one study (27) used mixture, while the rest of the fifteen studies used decoctions. Furthermore, 19 out of 22 studies described the components of these medicines. Regimens of chemotherapy were described in all studies. Among them, most of the studies used the FOLFOX regimen and XELOX/CAPOX regimen, while some used the IFL regimen, and irinotecan plus ramucirumab. Among the 16 studies that clarified toxicity assessments, 7 studies assessed outcomes by the toxicity criteria recommended by the WHO (14), 5 studies used the Guidelines for Clinical Research of New Chinese medicines (16), 2 studies used the National Cancer Institute Common Terminology Criteria for Adverse Events (15), 1 study used the National Cancer Institute Common Toxicity Criteria (43), and 1 study used the Recommendations of the European Society of Intensive Care Medicine (ESICM) Working Group on Abdominal Problems (44). Twenty studies that measured GI toxicity assessed by RR were subjected to meta-analysis, while 2 studies that adopted the rating scale (16) were analyzed separately. For some studies that reported the grades of GI toxicity, all grades were included except grade 0.

### Methodological Quality Assessment

**Figure 2:**
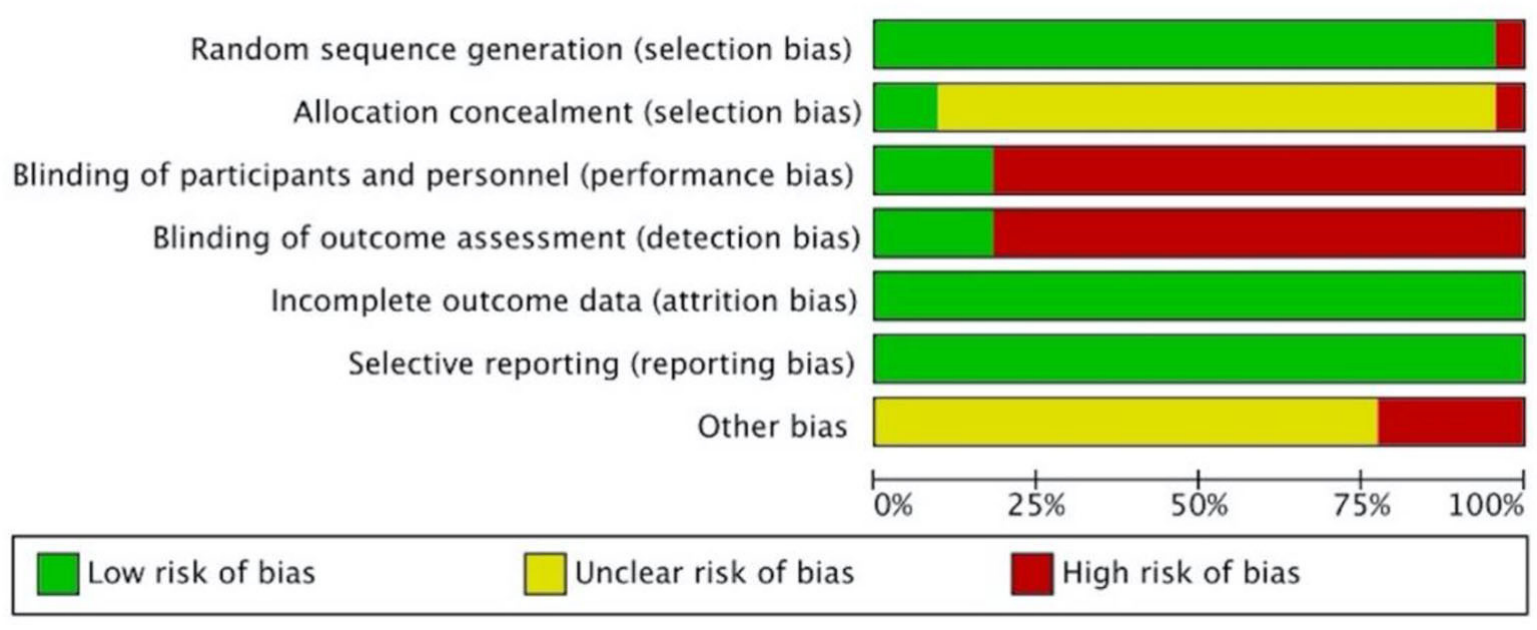
The overall risk of bias assessment using the Cochrane Collaboration's tool.

**Figure 3:**
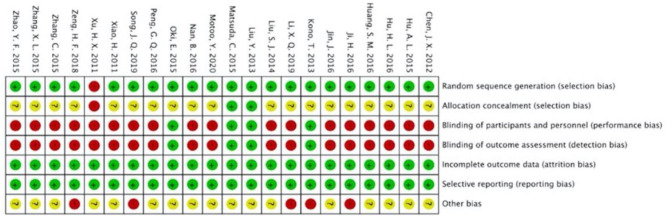
Risk of bias assessment by individual trials.

Figures 2, 3 present the overall RoB assessment and the methodological quality by individual selected studies, respectively. One study (21) did not perform random sequence generation and the rest of the 22 studies employed computer software or random number tables for randomization. However, only two studies (37, 39) described adequate allocation concealment. Performance bias and detection bias are the two primary sources of RoB. Participants in most studies were not blinded, as only four studies (36–39) conducted placebo controls and were judged as “low” RoB for performance and detection biases. All studies had a low RoB for incomplete outcome data or selective reporting due to the adequate description of dropouts. Five studies did not clarify toxicity assessments used for GI toxicity, which accounted for the high risk of other bias. The Jadad scores of the 22 included studies were in the range of 1–5, and the mean scores of studies were 3.27. Twenty-one of the included studies were of high quality (Jadad score ≥ 3).

### Analysis of the Overall Effects of Alleviating CIGI Toxicity

This meta-analysis was conducted for all grades of CIGI toxicity combined. The treatment group is favored when RR < 1 or MD < 0. A lower RR or MD represents a lower risk of CIGI toxicity. In our meta-analysis, a total of 20 studies with 1,509 patients were reported as RR. Data analysis for GI toxicity was stratified by studies with a double-blind design and studies without a double-blind design. Sixteen studies with 1,028 patients involved in this review did not adopt double-blind procedure, while 4 studies with 481 patients did. Data reported as MD for two studies (26, 27) were analyzed separately.

**Figure 4:**
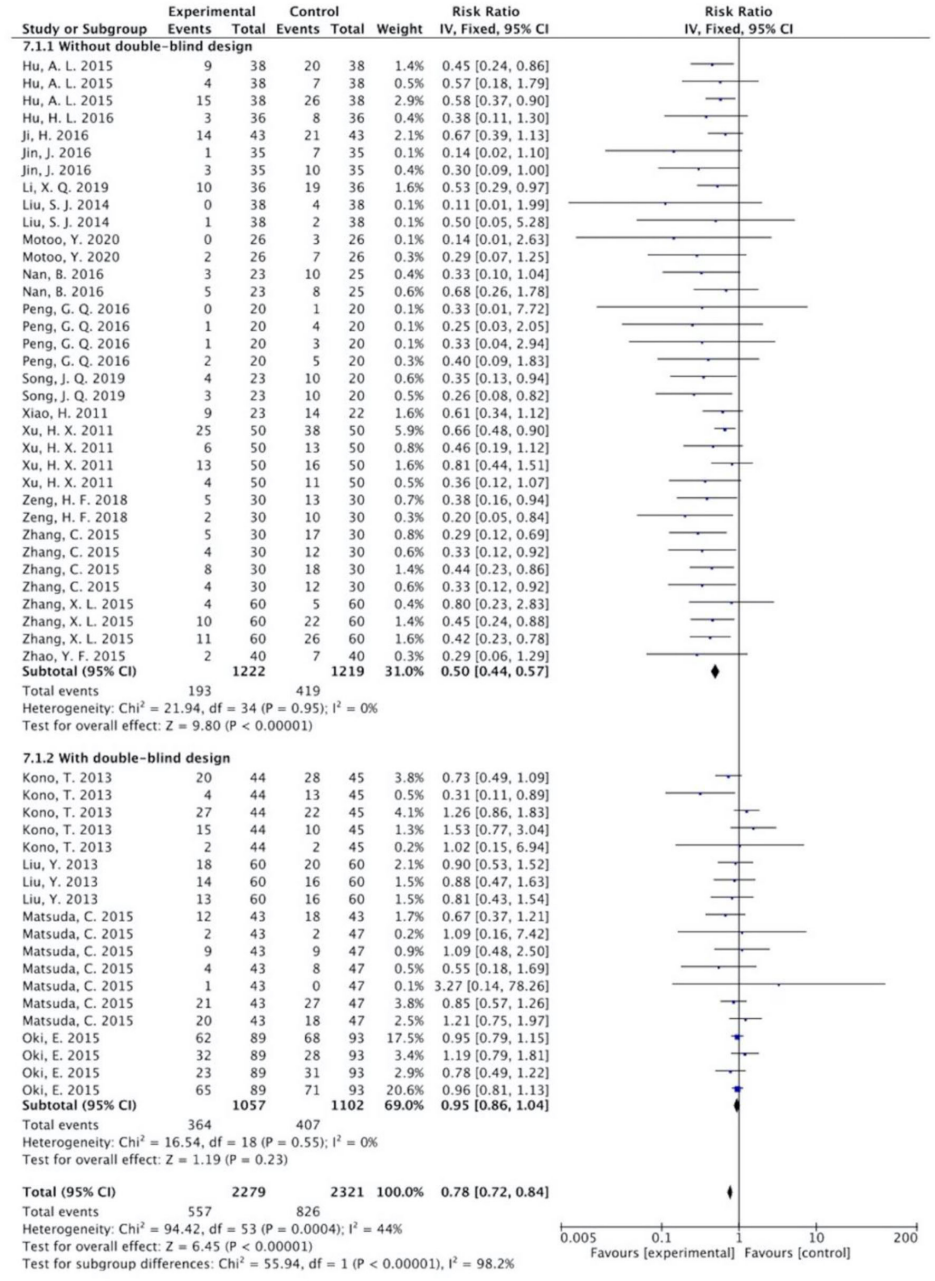
Overall effect of herbal medicine on chemotherapy-induced gastrointestinal toxicity reported as risk ratio.

**Figure 5:**
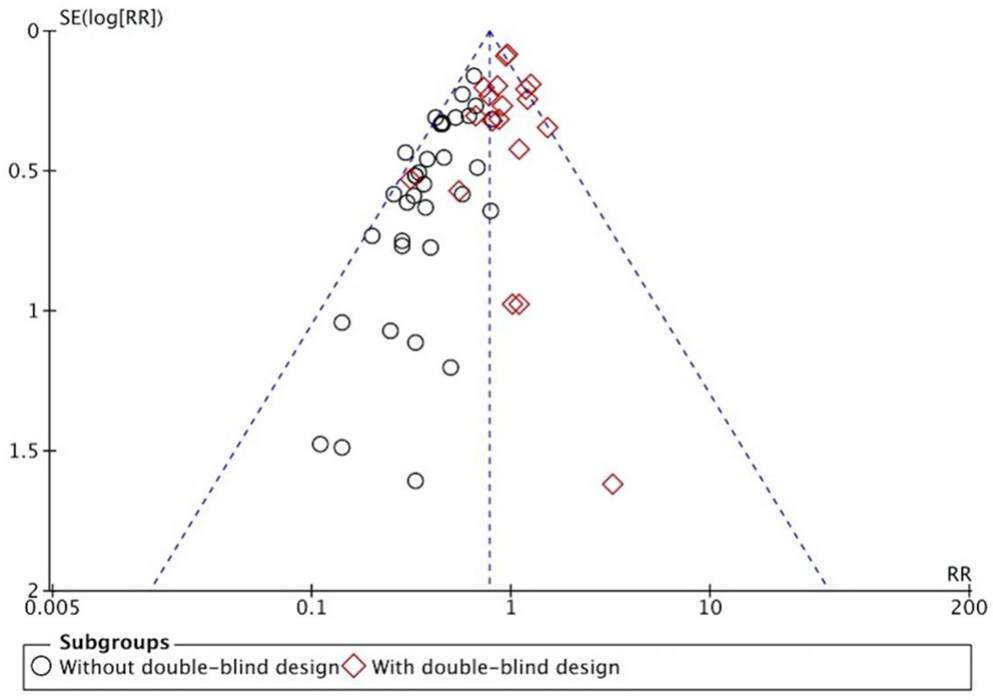
Funnel plot indicated potential publication bias.

**Figure 6:**
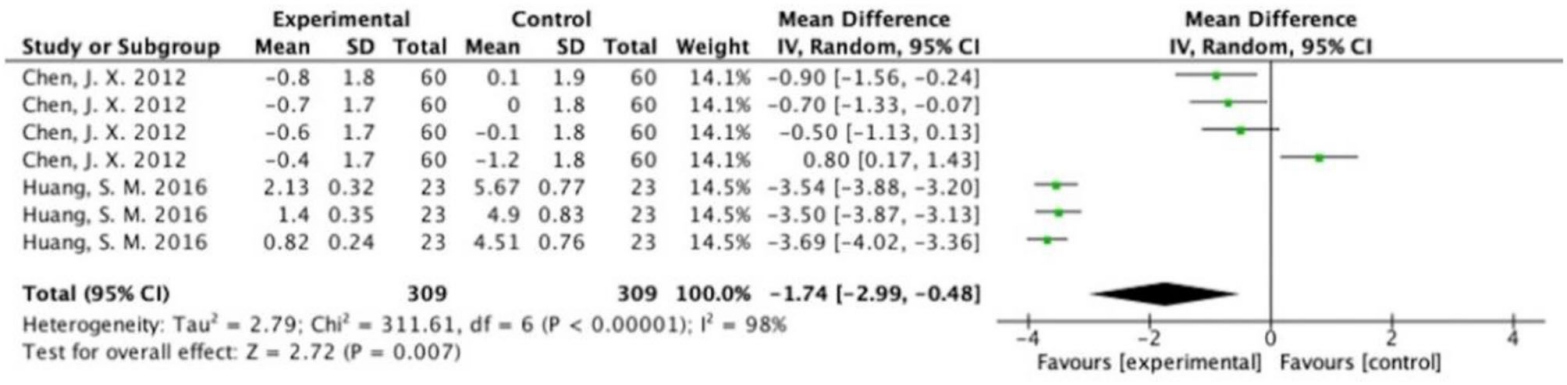
Overall effect of herbal medicine on chemotherapy-induced gastrointestinal toxicity reported as mean difference.

The overall results showed that, without differentiation of methodological quality, HM treatment combined with chemotherapy significantly alleviated CIGI toxicity, with an effect of 0.78 (95% CI = 0.72–0.84, *p* < 0.001, *I*^2^ = 44%) (Figure 4). The results demonstrated that the treatment groups significantly reduced CIGI toxicity compared to control groups in studies without a double-blind design, with an effect of 0.50 (95% CI = 0.44–0.57, *p* < 0.001, *I*^2^ = 0%); however, no statistically significant difference between treatment groups and control groups in studies with a double-blind design was found. The funnel plot was symmetrically distributed, suggesting that the risk of publication bias was relatively low in the included studies (Figure 5). A minor difference was observed in sensitivity analysis without essential change, indicating that the model is relatively stable. In the two studies that displayed data as mean difference, a statistically significant difference in favor of the treatment groups was shown, with an effect of −1.74 (95% CI = −2.99 to −0.48, *I*^2^ = 98%) (Figure 6).

### Sub-analysis

There were 17 studies that analyzed nausea and vomiting (21–25, 28–39), 13 studies that analyzed diarrhea (21, 24, 25, 29, 30, 32–34, 36–40), 7 studies that analyzed anorexia (26, 31–33, 35, 36, 38, 39), 3 studies that analyzed constipation (21, 36, 39), 5 studies that analyzed oral mucositis (21, 24, 29, 39, 41), 3 studies that analyzed abdominal pain (33, 39, 40), and 5 studies that analyzed abdominal distension (32, 33, 39, 40, 42). Sub-analysis of the above symptoms was stratified by studies with a double-blind design, and studies without a double-blind design.

**Figure 7:**
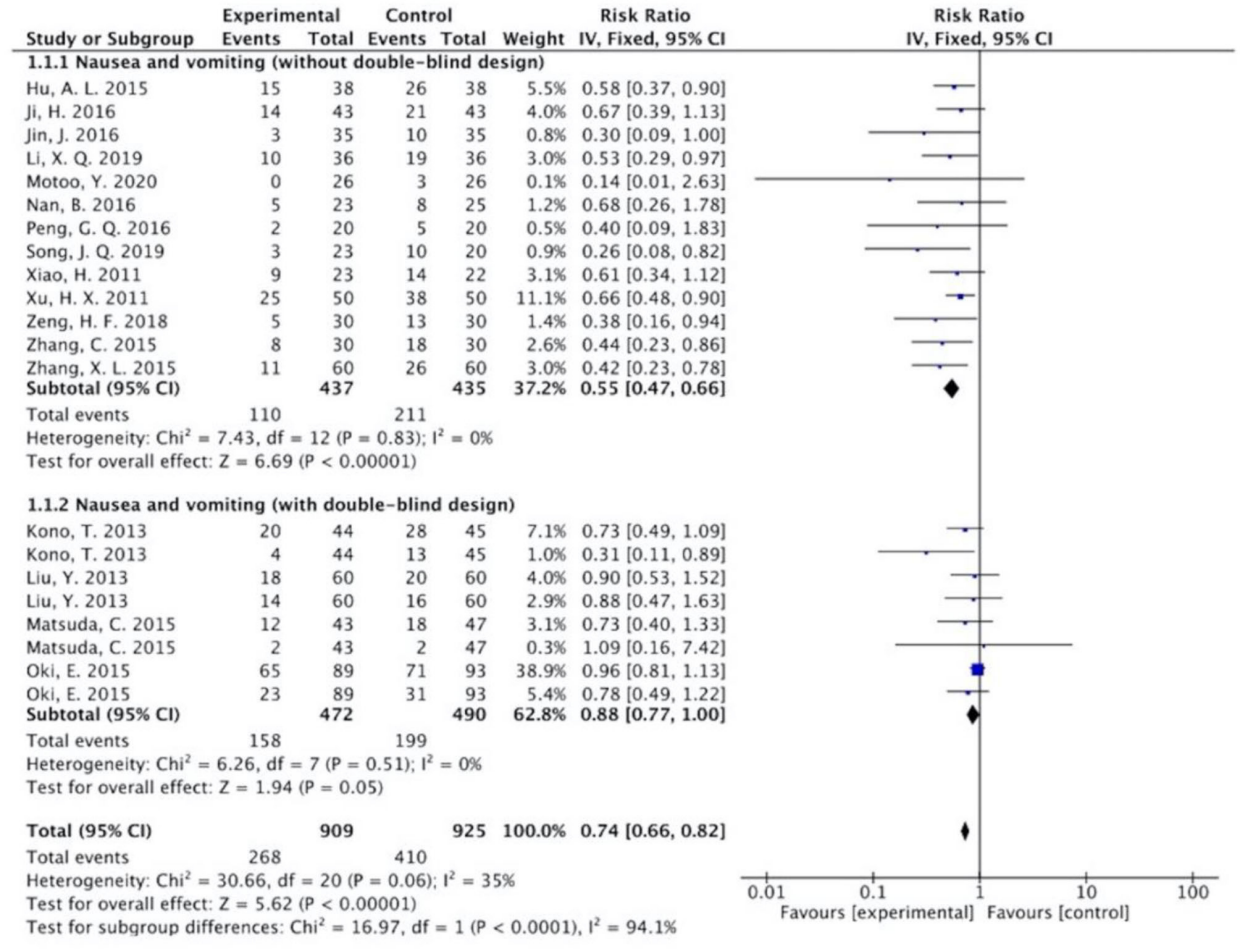
Sub-analysis on the effect of herbal medicine in nausea and vomiting in chemotherapy-induced toxicity.

**Figure 8:**
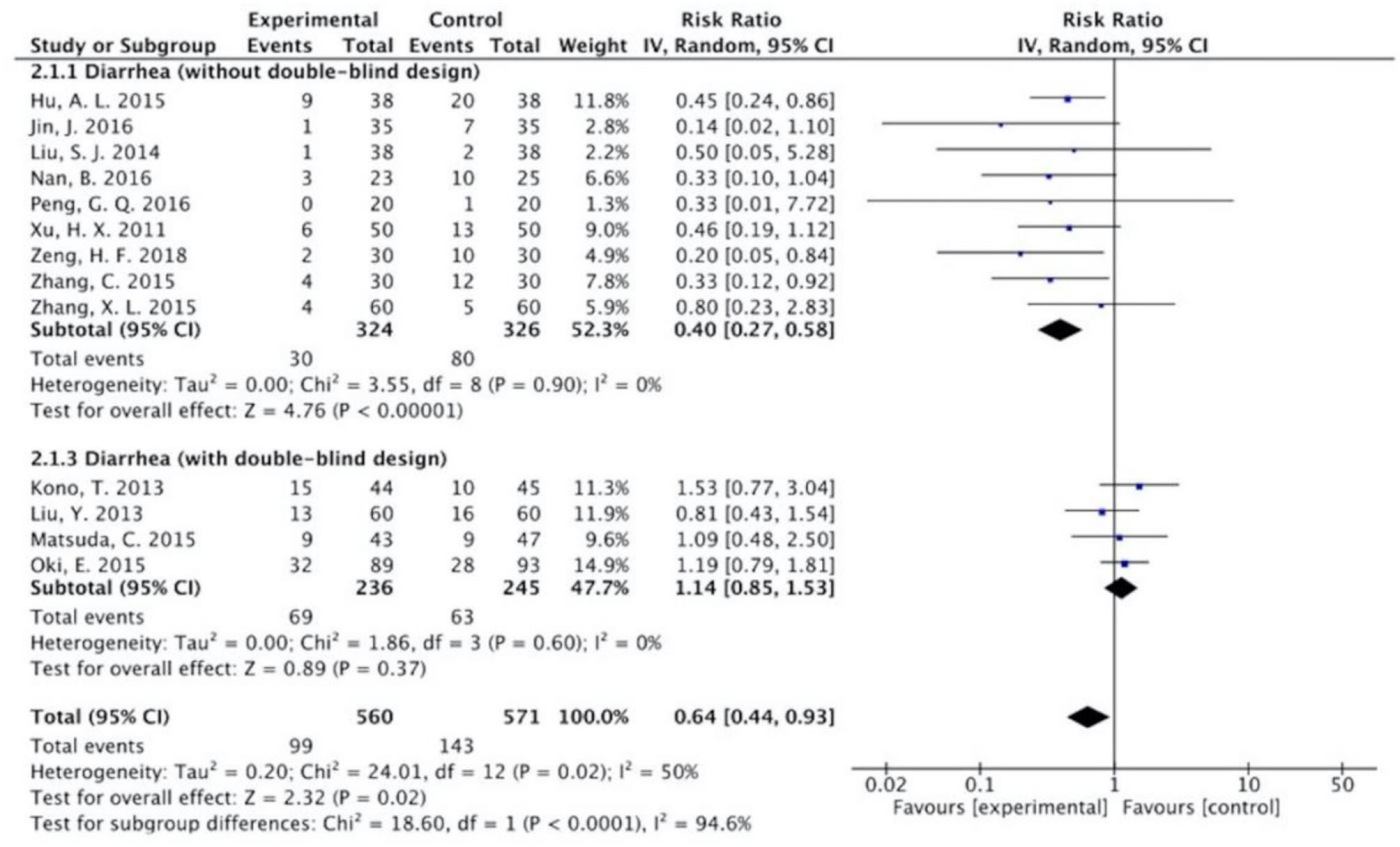
Sub-analysis on the effect of herbal medicine in diarrhea in chemotherapy-induced toxicity.

**Figure 9:**
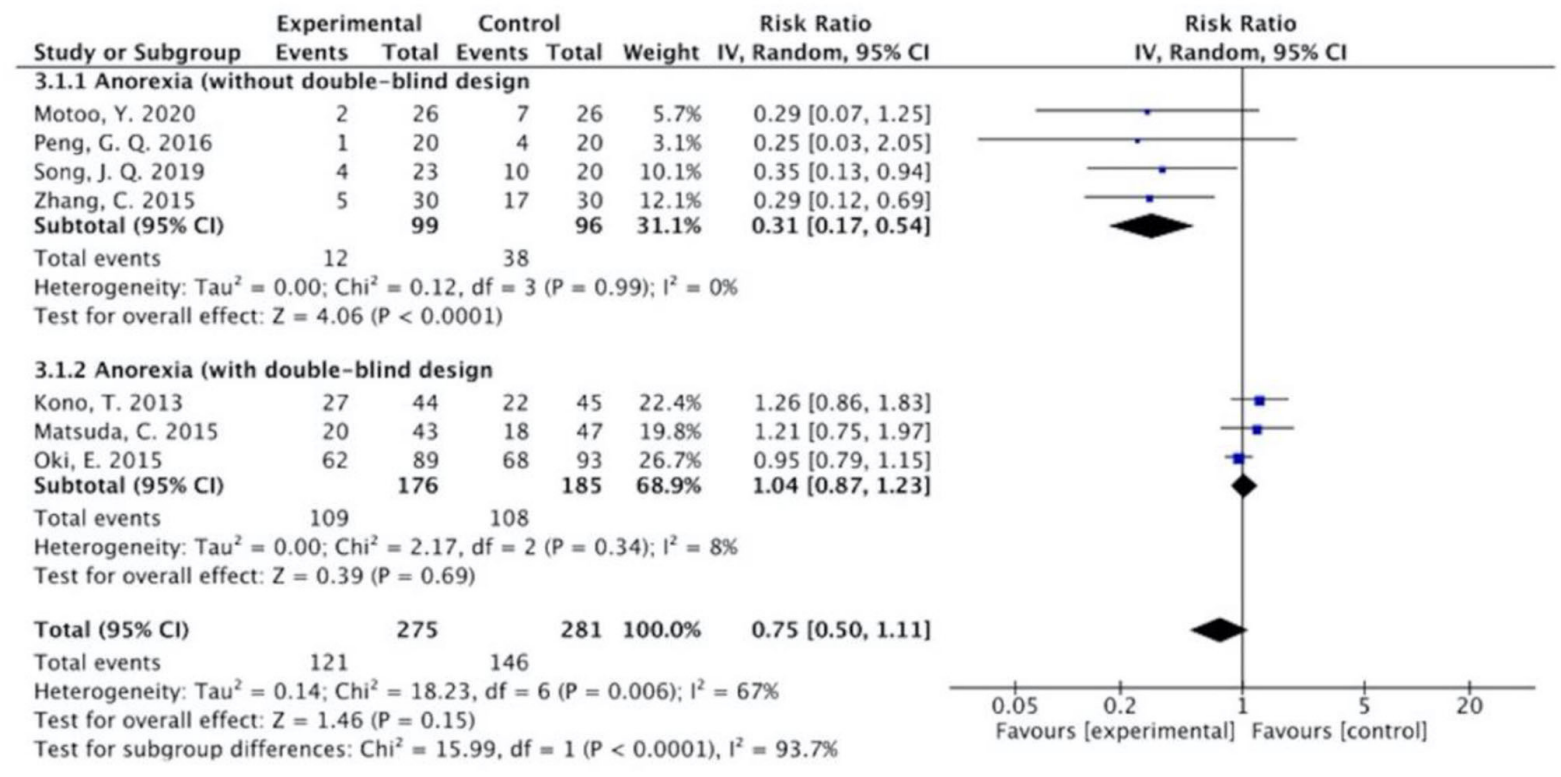
Sub-analysis on the effect of herbal medicine in anorexia in chemotherapy-induced toxicity.

**Figure 10:**
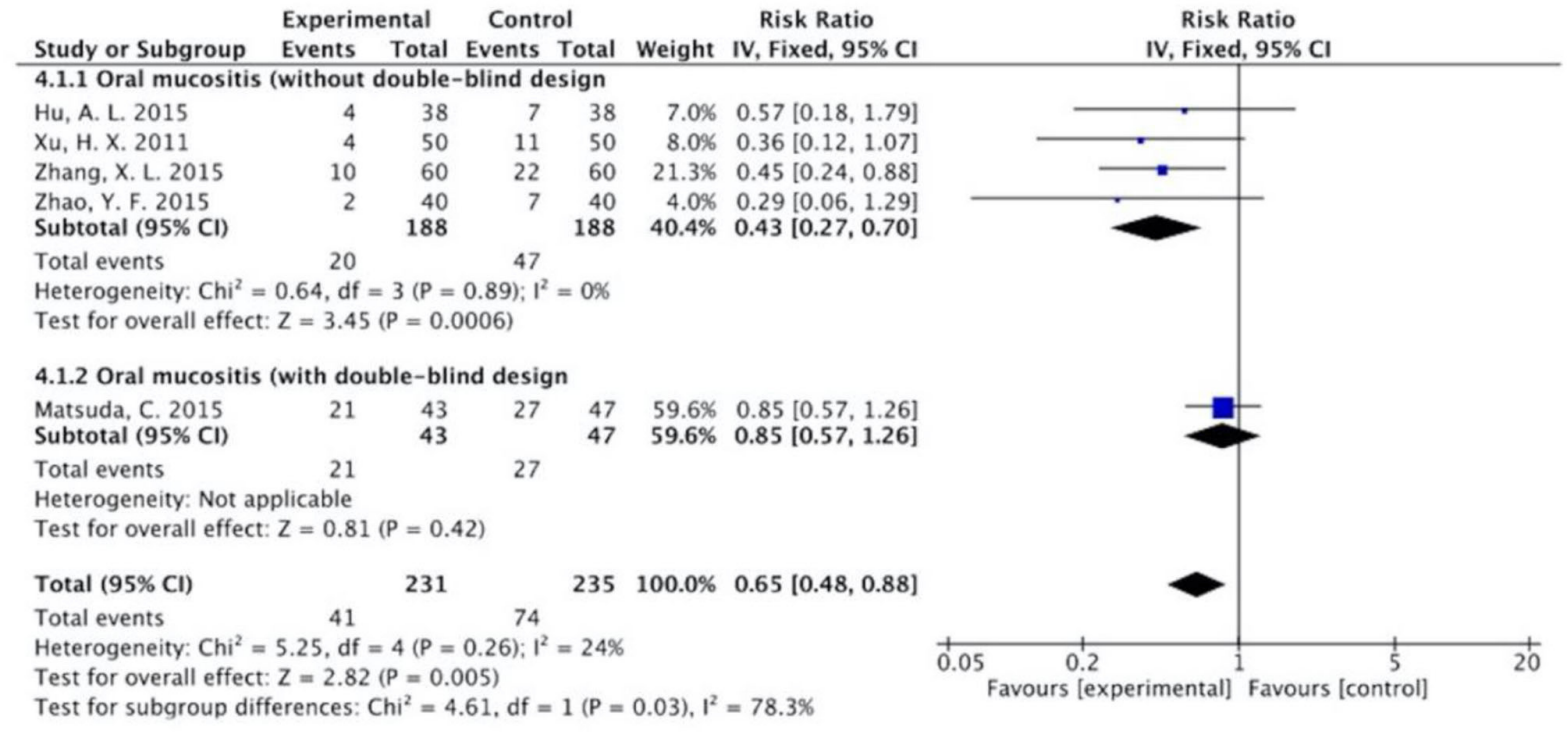
Sub-analysis on the effect of herbal medicine in oral mucositis in chemotherapy-induced toxicity.

**Figure 11:**
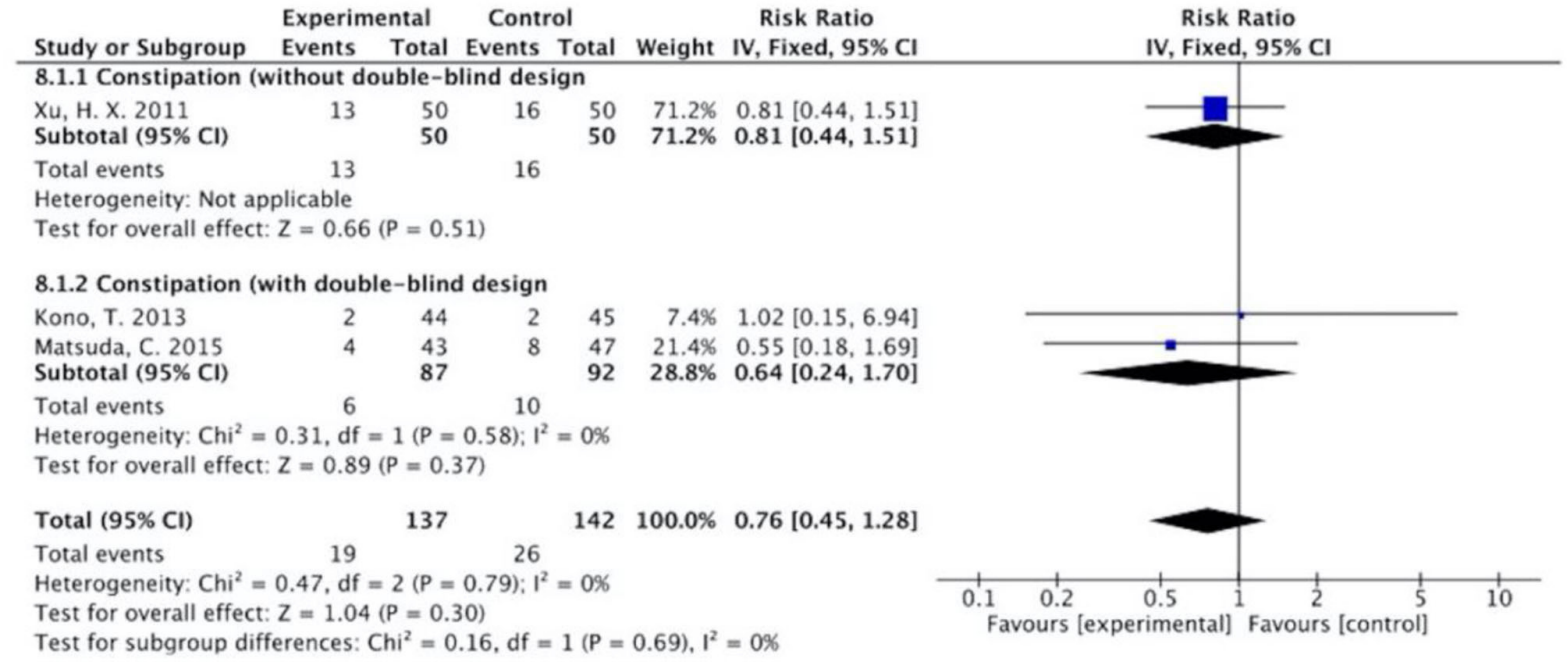
Sub-analysis on the effect of herbal medicine in constipation in chemotherapy-induced toxicity.

**Figure 12:**
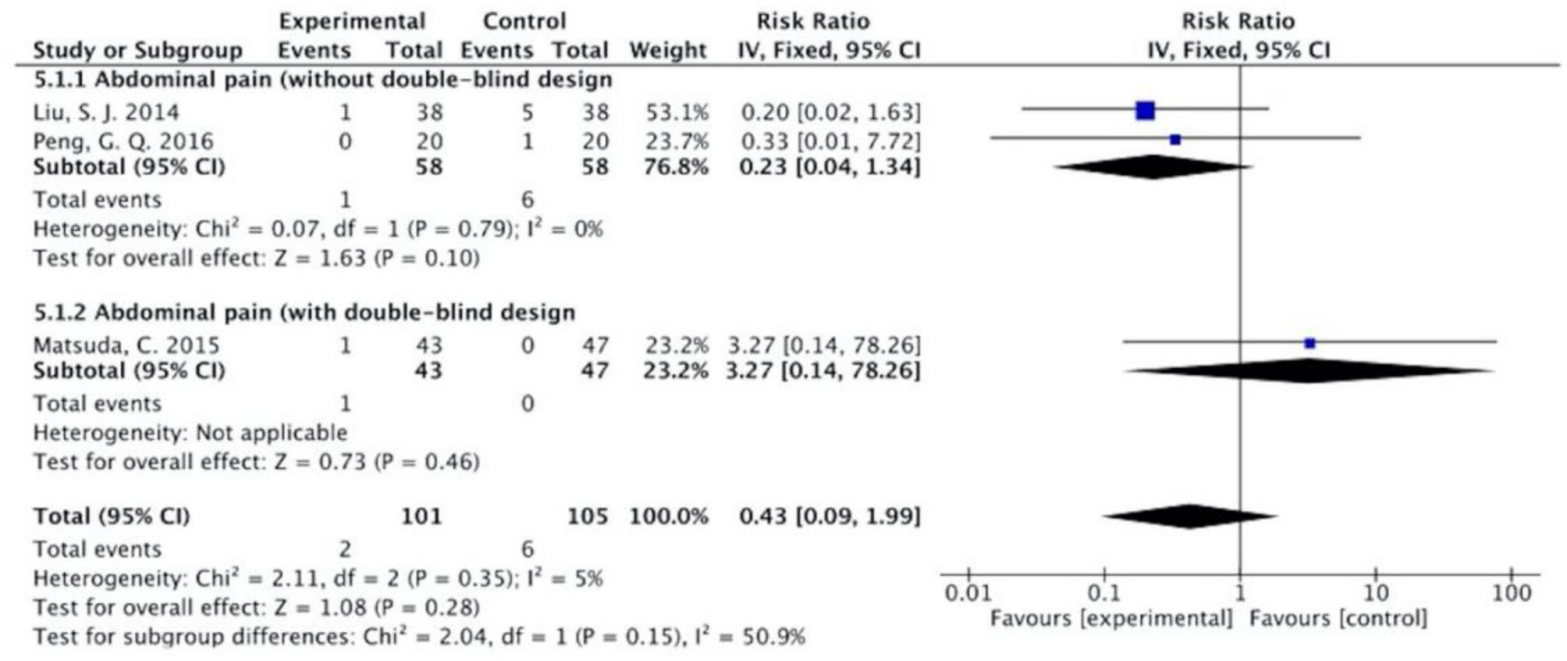
Sub-analysis on the effect of herbal medicine in abdominal pain in chemotherapy-induced toxicity.

**Figure 13:**
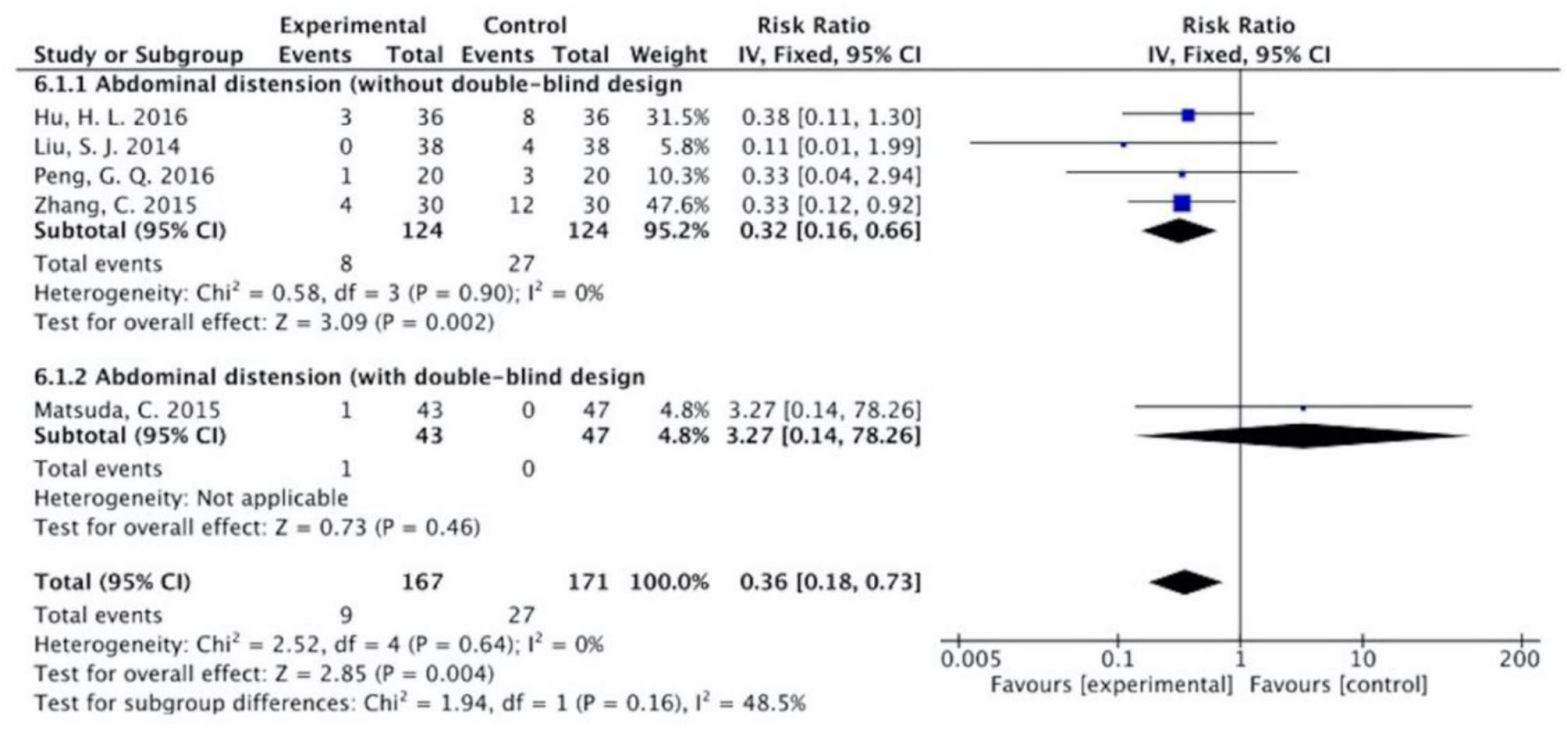
Sub-analysis on the effect of herbal medicine in abdominal distension in chemotherapy-induced toxicity.

The results (Figures 7–13) showed that for studies assessed without a double-blind design, the occurrence of nausea and vomiting, diarrhea, anorexia, oral mucositis, and abdominal distension significantly decreased in treatment groups. The RRs were 0.55 (95% CI = 0.47–0.66), 0.40 (95% CI = 0.27–0.58), 0.31 (95% CI = 0.17–0.54), 0.43 (95% CI = 0.27–0.70), and 0.32 (95% CI = 0.16–0.66). *I*^2^ was found at 0%. For studies with a double-blind design, the results of the occurrence of nausea and vomiting (RR = 0.88, 95% CI = 0.77–1.00), diarrhea (RR = 1.14, 95% CI = 0.85–1.53), and anorexia (RR = 1.04, 95% CI = 0.87–1.23) were not statistically significant.

Overall, without differentiation of methodological quality, the comparisons of the occurrence of the following symptoms between the two groups were statistically significant: nausea and vomiting (*p* < 0.001, RR = 0.74, 95% CI = 0.66–0.82, *I*^2^ = 35%), diarrhea (*P* = 0.02, RR = 0.64, 95% CI = 0.44–0.93, *I*^2^ = 50%), oral mucositis (*P* = 0.005, RR = 0.65, 95% CI = 0.48–0.88, *I*^2^ = 24%), and abdominal distension (*P* = 0.004, RR = 0.36, 95% CI = 0.18–0.73, *I*^2^ = 0%). No statistically significant results were identified in the occurrence of anorexia (*P* = 0.15, RR = 0.75, 95% CI = 0.50–1.11, *I*^2^ = 67%), constipation (*P* = 0.30, RR = 0.76, 95% CI = 0.45–1.28, *I*^2^ = 0%), and abdominal pain (*P* = 0.35, RR = 0.43, 95% CI = 0.09–1.99, *I*^2^ = 5%).

### Effects of Herbs in the Oral Administration Group

In multi-ingredient HM treatments, different combinations of the same herbs were used. In order to identify which herbs or herbal combinations that had the greatest contributions to the alleviation of CIGI toxicity, further sensitivity analyses of orally administered multi-ingredient HM interventions were conducted based on the composition of HM interventions.

The orally administered HMs contained 74 different herbs with an average of 10 herbs per treatment. The effects on the alleviation of CIGI toxicity of the herbs were presented at the level of the single herb, pair of herbs, and groups of three up to seven herbs. Thirty-two herbs were used in more than one study. The RR of the group of studies that contained each herb was calculated. Twenty-one of these herbs had significant RRs with low heterogeneity (*I*^2^ < 30%). The effects of these herbs (*n* = 21) that appeared in pairs, triplets, and higher-level combinations were assessed. All significant RR results were reported in Table 2, and only HMs with RR of low heterogeneity that were not greater than the total pooled RR (0.64 [0.55, 0.73]) were shown in the text.

**Table 2:** Effects of specific HMs on alleviation of chemotherapy-induced gastrointestinal toxicity: single HMs and combinations.

**Level**	**Herbal medicine (HM)**	**RR [95% CI]**	**N. stud. (Ref.)**	**N. part**.	***I*^2^**
1	*Schisandra chinensis* (Turcz.) (wu wei zi)	0.35 [0.23, 0.52]	2 [32, 35]	344	0
1	*Rheum officinale* Baill. (da huang)	0.36 [0.24, 0.54]	2 [32, 40]	392	0
1	*Taraxacum mongolicum* hand. Mazz. (pu gong ying)	0.36 [0.24, 0.54]	2 [28, 40]	392	0
1	*Aucklandia lappa* Decne. (mu xiang)	0.42 [0.24, 0.74]	3 [30, 33, 40]	408	0
1	*Oldenlandia diffusa* (Willd.) Roxb. (she she cao)	0.43 [0.31, 0.61]	3 [25, 28, 32]	371	0
1	*Amomum villosum* Lour. (sha ren)	0.44 [0.24, 0.80]	2 (23, 26)	256	0
1	*Curcuma zedoaria* (Berg.) Rosc. or C. phaeocaulis Val. (e zhu)	0.46 [0.23, 0.92]	2 [30, 33]	248	0
1	the pericarp of *Citrus reticulata* Blanco. (chen pi)	0.47 [0.37, 0.58]	6 [24, 28, 30, 32, 33, 35]	955	0
1	*Atractylodes macrocephala* Koidz. (bai zhu)	0.49 [0.39, 0.61]	8 [23, 24, 28, 30, 33–35, 41]	984	0
1	*Glycyrrhiza uralensis* Fisch. (gan cao)	0.51 [0.42, 0.63]	12 [23–25, 28, 30, 32–35, 37, 39, 41]	1,127	0
1	*Crataegus pinnatifida* Bge. Var. major N. E. Br. (shan zha)	0.53 [0.31, 0.88]	2 [28, 33]	205	0
1	*Coix lacryma-jobi* L. (yi ren)	0.54 [0.43, 0.69]	5 [23–25, 30, 41]	644	0
1	*Paeonia radix* alba. (bai shao)	0.55 [0.44, 0.70]	5 [30, 32, 34, 35, 37]	965	27
1	*Ligustrum lucidum* Ait. (nv zhen zi)	0.56 [0.38, 0.81]	3 [23, 25, 33]	318	0
1	*Sargentodoxa cuneata* (Oliv.) Rehd. et Wils (hong teng)	0.57 [0.32, 1.00]	2 [28, 40]	197	0
1	*Patrinia scabiosaefolia* Fisch. ex Link (bai jiang cao)	0.57 [0.36, 0.91]	2 [28, 40]	141	0
1	*Codonopsis pilosula* (Franch.). Nannf. (dang shen)	0.58 [0.49, 0.69]	7 [23–25, 28, 30, 32, 41]	280	0
1	*Solanum lyratum* Thunb. (bai ying)	0.60 [0.40, 0.90]	2 [23, 25]	158	0
1	*Angelica sinensis* (Oliv.) Diels. (dang gui)	0.63 [0.48, 0.83]	5 [30, 33–35, 37]	840	0
1	*Pinellia ternata* (Thunb.) Breit. (ban xia)	0.76 [0.62, 0.92]	5 [23, 25, 30, 33, 39]	540	0
1	*Astragalus membranaceus* (Fisch.) Bge. (huang qi)	0.76 [0.65, 0.90]	8 [16, 18, 21, 26, 28, 30, 32, 34]	1533	0
2	R. officinale + T. mongolicum	0.36 [0.24, 0.54]	2 [25, 33]	392	0
2	C. zedoaria + A. lappa	0.46 [0.23, 0.92]	2 [23, 33]	248	0
2	G. uralensis + C. reticulata	0.47 [0.37, 0.58]	6 [17, 21, 23, 25, 26, 28]	995	0
2	G. uralensis + A. macrocephala	0.49 [0.39, 0.61]	8 [23, 24, 28, 30, 33–35, 41]	238	0
2	G. uralensis + C. pilosula	0.51 [0.42, 0.63]	7 [22–24, 28, 30, 32, 41]	1073	0
2	G. uralensis + P. radix	0.55 [0.44, 0.70]	5 [23, 25, 27, 28, 30]	920	27
2	A. sinensis + G. uralensis	0.63 [0.48, 0.83]	5 [30, 33–35, 37]	840	0
2	G. uralensis + A. membranaceus	0.73 [0.62, 0.86]	8 [22, 23, 28, 33, 35, 37, 39, 41]	403	2
2	G. uralensis + P. ternata	0.76 [0.62, 0.92]	5 [22, 23, 30, 33, 39]	1,040	0
3	A. sinensis + G. uralensis + A. macrocephala	0.37 [0.24, 0.58]	4 [30, 33–35]	480	0
3	G. uralensis + C. reticulata + P. radix	0.38 [0.27, 0.54]	3 [30, 32, 35]	440	0
3	G. uralensis + C. reticulata + C. pilosula	0.48 [0.38, 0.61]	4 [24, 28, 30, 32]	691	0
3	G. uralensis + A. macrocephala + P. ternata	0.48 [0.31, 0.73]	3 [23, 30, 33]	328	0
3	G. uralensis + A. macrocephala + A. membranaceus	0.48 [0.33, 0.69]	5 [23, 28, 33, 35, 41]	461	0
3	G. uralensis + O. diffusa + C. pilosula	0.49 [0.37, 0.66]	3 [22, 28, 32]	371	0
3	G. uralensis + A. macrocephala + C. reticulata	0.51 [0.40, 0.66]	5 [24, 28, 30, 33, 35]	715	0
3	G. uralensis + A. macrocephala + C. pilosula	0.53 [0.42, 0.68]	5 [23, 24, 28, 30, 41]	603	0
3	G. uralensis + C. lacryma + C. pilosula	0.54 [0.43, 0.69]	5 [22–24, 30, 41]	644	0
3	G. uralensis + A. membranaceus + C. pilosula	0.59 [0.42, 0.81]	4 [22, 23, 28, 41]	283	0
3	A. sinensis + G. uralensis + P. radix	0.66 [0.50, 0.88]	4 [30, 34, 35, 37]	680	22
3	A. sinensis + G. uralensis + A. membranaceus	0.74 [0.54, 1.01]	3 [33, 35, 37]	624	0
3	G. uralensis + A. membranaceus + P. ternata	0.78 [0.64, 0.96]	4 [22, 23, 33, 39]	944	0
4	G. uralensis + C. reticulata + P. radix + S. chinesis	0.35 [0.23, 0.52]	2 [32, 35]	344	0
4	A. sinensis + G. uralensis + A. macrocephala + P. radix	0.38 [0.23, 0.62]	3 [30, 34, 35]	320	0
4	G. uralensis + C. reticulata + P. radix + C. pilosula	0.39 [0.27, 0.57]	2 [30, 32]	336	0
4	A. sinensis + G. uralensis + A. macrocephala + C. reticulata	0.40 [0.23, 0.69]	3 [30, 33, 35]	360	0
4	G. uralensis + C. reticulata + O. diffusa + C. pilosula	0.43 [0.31, 0.61]	2 [28, 32]	285	0
4	G. uralensis + A. macrocephala + C. reticulata + A. membranaceus	0.48 [0.29, 0.77]	3 [28, 33, 35]	309	0
4	G. uralensis + A. macrocephala + C. lacryma + C. pilosula	0.52 [0.39, 0.68]	4 [23, 24, 30, 41]	558	0
4	G. uralensis + A. macrocephala + C. reticulata + C. pilosula	0.54 [0.41, 0.72]	3 [24, 28, 30]	451	0
4	G. uralensis + A. macrocephala + A. membranaceus + C. pilosula	0.54 [0.36, 0.82]	3 [23, 28, 41]	197	0
4	G. uralensis + L. lucidum + A. membranaceus + P. ternata	0.56 [0.38, 0.81]	3 [22, 23, 33]	318	0
4	G. uralensis + C. lacryma + A. membranaceus + C. pilosula	0.57 [0.39, 0.84]	3 [22, 23, 41]	238	0
4	G. uralensis + C. lacryma + P. ternata + C. pilosula	0.58 [0.41, 0.82]	3 [22, 23, 30]	254	0
4	G. uralensis + A. membranaceus + O. diffusa + C. pilosula	0.64 [0.43, 0.96]	2 [24, 28]	131	0
4	A. sinensis + G. uralensis + A. membranaceus + P. radix	0.80 [0.58, 1.11]	2 [35, 37]	464	0
5	A. sinensis + G. uralensis + A. macrocephala + C. reticulata + A. membranaceus	0.30 [0.13, 0.67]	2 [33, 35]	264	0
5	A. sinensis + G. uralensis + A. macrocephala + C. reticulata + P. radix	0.42 [0.22, 0.81]	2 [30, 35]	200	0
5	G. uralensis + A. macrocephala + L. lucidum + A. membranaceus + P. ternata	0.47 [0.28, 0.79]	2 [23, 33]	232	0
5	G. uralensis + A. macrocephala + C. lacryma + A. membranaceus + C. pilosula	0.48 [0.27, 0.85]	2 [23, 41]	152	0
5	G. uralensis + A. macrocephala + C. lacryma + P. ternata + C. pilosula	0.52 [0.32, 0.83]	2 [23, 30]	168	0
5	G. uralensis + A. macrocephala + C. lacryma + C. reticulata + C. pilosula	0.53 [0.39, 0.72]	2 [24, 30]	406	0
5	G. uralensis + A. macrocephala + C. reticulata + A. membranaceus + C. pinnatifida	0.53 [0.31, 0.88]	2 [28, 33]	205	0
5	G. uralensis + A. macrocephala + C. reticulata + P. scabiosaefolia + C. pilosula	0.57 [0.36, 0.91]	2 [28, 30]	141	0
7	A. villosum + A. sinensis + G. uralensis + A. macrocephala + C. reticulata + A. lappa + P. ternata	0.44 [0.24, 0.80]	2 [30, 33]	256	0
7	G. uralensis + S. lyratum + C. lacryma + L. lucidum + A. membranaceus + P. ternata + C. pilosula	0.60 [0.40, 0.90]	2 [22, 23]	158	0

*RR, risk ratio; N. part., number of participants; N. stud., number of studies; CI, confidence interval; Ref., reference*.

The more frequently used herbs in the HM treatments were: *Glycyrrhiza uralensis* Fisch. (gan cao) (*n* = 12), *Atractylodes macrocephala* Koidz. (bai zhu) (*n* = 8), *Astragalus membranaceus* (Fisch.) Bge. (huang qi) (*n* = 8), *Codonopsis pilosula* (Franch.) Nannf. (dang shen) (*n* = 7), and the pericarp of *Citrus reticulata* Blanco. (chen pi) (*n* = 6).

### Level 1: Single HMs

Of the 21 plants shown at the Level 1 analysis, two did not combined with another plant. They were *Aucklandia lappa Decne*. (mu xiang) (*n* = 3) (RR 0.42 [0.24, 0.74], *I*^2^ = 0%), and *Glycyrrhiza uralensis Fisch*. (gan cao) (*n* = 12) (RR 0.51 [0.42, 0.63], *I*^2^ = 0%). The rest of the plants associated with at least one other plant.

### Level 2: Pairs of HMs

Twenty-one plants were paired with other plants from Level 1, and 9 pairs were generated. Seven pairs had lower RRs when compared with the total pool RR for orally administered HM intervention. *Rheum officinale* + *Taraxacum mongolicum* (*n* = 2) (RR 0.36 [0.24, 0.54], *I*^2^ = 0%) had the lowest RR, followed by *Curcuma zedoaria* + *Arctium lappa* (*n* = 2) (RR 0.46 [0.23, 0.92], *I*^2^ = 0%), and *G. uralensis* + *C. reticulata* (*n* = 6) (RR 0.47 [0.37, 0.58], *I*^2^ = 0%). The most frequent pairs were *G. uralensis* + *A. macrocephala* (*n* = 8) (RR 0.49 [0.39, 0.61], *I*^2^ = 0%) and *G. uralensis* + *C. pilosula* (*n* = 7) (RR 0.51 [0.42, 0.63], *I*^2^ = 0%).

### Level 3: Combinations of Three HMs

Compared with the total pool RR, 10 combinations of three plants presented lower RRs. The most frequent combinations were *G. uralensis* + *Coix lacryma* + *C. pilosula* (*n* = 5) (RR 0.54 [0.43, 0.69], *I*^2^ = 0%), *G. uralensis* + *A. macrocephala* + *C. reticulata* (*n* = 5) (RR 0.51 [0.40, 0.66], *I*^2^ = 0%), *G. uralensis* + *A. macrocephala* + *Astragalus membranaceus* (*n* = 5) (RR 0.48 [0.33, 0.69], *I*^2^ = 0%), and *G. uralensis* + *A. macrocephala* + *C. pilosula* (*n* = 5) (RR 0.53 [0.42, 0.68], *I*^2^ = 0%). The combination of *G. uralensis* + *C. reticulata* + *Paeonia radix* (*n* = 2) had the lowest RR (0.38 [0.27, 0.54], *I*^2^ = 0%).

### Level 4: Combinations of Four HMs

Compared with the total pool RR, 13 combinations of four plants showed lower RRs. *G. uralensis* + *A. macrocephala* + *C. lacryma* + *C. pilosula* (*n* = 4) (RR 0.52 [0.39, 0.68], *I*^2^ = 0%) was the most frequent combination. *G. uralensis* + *C. reticulata* + *P. radix* + *Schisandra chinensis* (*n* = 2) had the lowest RR (0.35 [0.23, 0.52], *I*^2^ = 0%), followed by *Angelica sinensis* + *G. uralensis* + *A. macrocephala* + *P. radix* (*n* = 3) (0.38 [0.23, 0.62], *I*^2^ = 0%).

### Level 5: Combinations of Five HMs

Compared with the total pool RR, RRs of the eight combinations of five plants were lower. All of the combinations appeared in two studies. The combination of *A. sinensis* + *G. uralensis* + *A. macrocephala* + *C. reticulata* + *A. membranaceus* had the lowest RR (0.30 [0.13, 0.67], *I*^2^ = 0%), followed by *A. sinensis* + *G. uralensis* + *A. macrocephala* + *C. reticulata* + *P. radix* (RR 0.42 [0.22, 0.81], *I*^2^ = 0%), and *G. uralensis* + *A. macrocephala* + *Ligustrum lucidum* + *A. membranaceus* + *Pinellia ternata* (RR 0.47 [0.28, 0.79], *I*^2^ = 0%).

### Level 7: Combinations of Seven HMs

Compared with the total pool RR, RRs of the two combinations of seven plants were lower. They were *G. uralensis* + *Solanum lyratum* + *C. lacryma* + *L. lucidum* + *A. membranaceus* + *P. ternata* + *C. pilosula* (*n* = 2) (RR 0.60 [0.40, 0.90], *I*^2^ = 0%), and *Amomum villosum* + *A. sinensis* + *G. uralensis* + *A. macrocephala* + *C. reticulata* + *A. lappa* + *P. ternata* (*n* = 2) (RR 0.44 [0.24, 0.80], *I*^2^ = 0%).

### Herbal Medicines With Consistent Results at Multiple Levels

Plants that showed significant RR results that were not greater than the pool total RR, with heterogeneity < 30% at multiple levels, were identified and selected for further research. Five plants were shown at all six levels. They were *G. uralensis, C. reticulata, A. sinensis, C. pilosula*, and *A. macrocephala*. Therefore, a clinical benefit for CIGI toxicity was suggested when these five plants were included in HM interventions.

### Potential Synergistic Effects of HMs

Eleven combinations of plants presented lower RRs compared with those of the plants from Level 1. The following four triplets were included: G. uralensis + C. reticulata + P. radix, *A. sinensis* + *G. uralensis* + *A. macrocephala, G. uralensis* + *A. macrocephala* + *P. ternata*, and *G. uralensis* + *A. macrocephala* + *A. membranaceus*; three combinations of four plants: *G. uralensis* + *C. reticulata* + *P. radix* + *C. pilosula, A. sinensis* + *G. uralensis* + *A. macrocephala* + *C. reticulata*, and *A. sinensis* + *G. uralensis* + *A. macrocephala* + *P. radix*; four combinations of five plants: *A. sinensis* + *G. uralensis* + *A. macrocephala* + *C. reticulata* + *P. radix, G. uralensis* + *A. macrocephala* + *C. lacryma* + *A. membranaceus* + *C. pilosula, A. sinensis* + *G. uralensis* + *A. macrocephala* + *C. reticulata* + *A. membranaceus*, and *G. uralensis* + *A. macrocephala* + *L. lucidum* + *A. membranaceus* + *P. ternata*. Of these, the combination of *A. sinensis* + *G. uralensis* + *A. macrocephala* + *C. reticulata* + *A. membranaceus* (*n* = 2) had the lowest RR (0.30 [0.13, 0.67], *I*^2^ = 0%), followed by *A. sinensis* + *G. uralensis* + *A. macrocephala* (*n* = 4) (RR 0.37 [0.24, 0.58], *I*^2^ = 0%), *G. uralensis* + *C. reticulata* + *P. radix* (*n* = 3) (RR 0.38 [0.27, 0.54], *I*^2^ = 0%), and *A. sinensis* + *G. uralensis* + *A. macrocephala* + *P. radix* (*n* = 3) (RR 0.38 [0.23, 0.62], *I*^2^ = 0%).

## Discussion

This review and meta-analysis evaluated 22 studies on the combination of the HMs with chemotherapy as an intervention to manage CIGI toxicity in patients with CRC. Evidence was found of an association between HMs and relief from CIGI symptoms. In our meta-analysis, the effects of HMs on nausea and vomiting and diarrhea are generally in line with previous meta-analyses (10–13); however, none of the included studies performed the placebo-controlled, double-blind procedure. In contrast to the previous meta-analyses, our study reported no statistical significance between chemotherapy plus HMs and comparators regarding the effects on anorexia in patients with CRC, while a previous analysis conducted by Zhong et al. showed significant effects favoring HMs (11). The discrepancies of results may arise mainly from the difference in sample size and study quality. Zhong's meta-analysis included only three studies without a placebo-controlled, double-blind design, which likely caused significant bias and influenced the results.

No previous meta-analysis on constipation, oral mucositis, abdominal pain, and abdominal distension that compared the combination of HMs with chemotherapy over the same chemotherapy regimen in patients with CRC was identified. Chung et al. assessed the effects of TCM in conjunction with conventional medicine or chemotherapy for alleviating constipation in patients with cancer by investigating seven RCTs (45); however, data were not pooled because the definition of significant relief among included studies that were not the same. Yuan et al. conducted a meta-analysis evaluating the efficacy of TCM for oral mucositis (46). The study included 8 RCTs with 694 patients and compared the effects of the combination of TCM and concurrent chemoradiotherapy with chemoradiotherapy. Significant reductions in the severity of oral mucositis were shown (RR = 0.52, 95% CI = 0.43–0.64, *p* < 0.001); however, all studies included in this meta-analysis showed low methodological quality, and the treatment involved both chemotherapy and radiotherapy. CRC was not specified in any of the included studies by Yuan et al. (46).

Our present meta-analysis identified that among studies without a double-blind design, the HMs groups significantly alleviated symptoms of CIGI toxicity, including nausea and vomiting, diarrhea, anorexia, oral mucositis, and abdominal distension. However, among studies with a double-blind design, no statistical differences were found between HMs groups and control groups in all GI symptoms. Our sensitivity analysis between studies with and without a double-blind design showed a statistically significant difference for overall GI toxicity, nausea and vomiting, diarrhea, anorexia, and oral mucositis. The observed differences demonstrated that trials are more likely to show the advantage of the combination of HMs with chemotherapy over chemotherapy alone if a double-blind design is not employed. This difference may arise from a combination of response bias and placebo effect if the outcomes were patient-reported (47). Patients in the intervention groups may have high expectations for HMs treatment and therefore lower the severity of symptoms on self-rating scales. Chen et al. commended that in subjective outcome measurements, such as for the symptom of nausea, may be influenced by the lack of blinding, and a non-specific effect is likely to be produced (20). Moreover, if the outcomes in trials were assessed by non-blinded investigators, the outcomes could also be less reliable and less objective, arising from a favorable reporting toward the intervention groups (20).

Moreover, four studies (36–39) on diarrhea and three studies (36, 38, 39) on anorexia were accessed among the studies with a double-blind design. Positive effects favoring the control groups were shown in the occurrence of both diarrhea and anorexia, suggesting that the prescribed HM combinations including TJ-107 (Goshajinkigan), Guilongtongluofang, and TJ-14 (Hangeshashinto) did not exert a positive effect on alleviating diarrhea or anorexia. It is worth noting that, all of these studies investigated diarrhea or anorexia as secondary outcomes, hence the possibility that the combination therapy caused the CIGI toxicity could not be ruled out. Well-designed RCTs should be further conducted to provide more evidence.

Herbal medicine treatments are composed of a variety of herbs used in different combinations and forms. Although the treatments vary among studies, the herbs are commonly used in multiple studies (20). The following five herbs that had significant pooled RRs, without heterogeneity, were consistently present at multiple levels of combination: *G. uralensis* (*n* = 12), *C. reticulata* (*n* = 6), *A. sinensis* (*n* = 5), *C. pilosula* (*n* = 7), and *A. macrocephala* (*n* = 8). These herbs were thus considered to show consistent effects on alleviating CIGI toxicity in multiple combinations. Significant alleviation of GI toxicity was also found for other herbs and herb combinations; however, low frequency of the herbs provided insufficient information to assess their effects. For example, *A. villosum* (*n* = 2), *P. ternata* (*n* = 5), and *A. lappa* (*n* = 3) appeared in a subgroup that showed the greatest reduction in GI toxicity at Level 7. It is possible that these herbs also contributed to the results although the subgroup included *G. uralensis, C. reticulata, A. membranaceus, and A. sinensis*. Therefore, it is essential to note that the herbs selected in the final analyses are not the only herbs that had effects on alleviating CIGI toxicity. Instead, they had consistent effects in multiple combinations.

Although the five herbs are commonly used in combination, there is insufficient information about the efficacy of single herbs in treating CIGI toxicity in patients with CRC. Studies of these five herbs related to CIGI or GI toxicity reduction on experimental models in animals were then explored, which may give an explanation of the effects shown in the meta-analyses to a certain extent. Zhou et al. reported that *Radix Codonopsi Polysaccharide* significantly decreased the diarrhea scores and the levels of TNF-α, IL-1β, and IL-6 in mice with 5-FU-induced GI mucositis when compared with positive control groups (48). Chen and Zhang investigated the effects of the *C. pilosula* and *A. macrocephala* on promoting growth and differentiation of small intestine epithelial IEC-6 cells in normal rats, found that the combination of *C. pilosula* and *A. macrocephala* stimulated IEC-6 cells growth and differentiation more evidently than these HMs used singly (49). A study reported by He showed that water extractive of *C. reticulata* significantly increased the contraction of small intestine smooth muscle in rats with GI motility disorder (50). Du et al. concluded that *A. sinensis* improved the mucosal atrophy, increased the secretion of colonic mucus, and thus had a statistically significant effect on improving constipation (51).

Potential synergistic effects of the HM combination of three of the five herbs were explored. A HM called Si-Jun-Zi decoction (SJZD), which contains *C. pilosula, G. uralensise*, and *A. macrocephala*, is used for treating GI disorders (52). Evidence for an association between SJZD and CIGI symptoms relieve was found. Ni assessed the effects of FOLFOX-7 chemotherapy combined application of modified SJZD on CIGI toxicity (anorexia, abdominal distension, and loose stool) in 70 patients with spleen-stomach Qi deficiency syndrome, and statistically significant alleviation of CIGI toxicity was shown (53). Li et al. conducted a meta-analysis including 8 studies with 483 eligible patients, which compared the effects of chemotherapy combined with SJZD and chemotherapy-alone in patients with CRC (54). They concluded that SJZD showed significant alleviations in CIGI toxicity including nausea and vomiting, and diarrhea, compared to chemotherapy alone. Experimental models in animals have also revealed the efficacy of SJZD on alleviating GI toxicity. Zheng et al. assessed the effects of aqueous extracts of herbs in SJZD, including *G. uralensis, C. pilosula*, and *A. macrocephala* on contractile of isolated rat gastric muscle strips (55). They found that *G. uralensis, C. pilosula*, and *A. macrocephala* enhanced longitudinal muscle tension in strips from the gastric body, *G. uralensis* and *C. pilosula* increased the motility index of pyloric circular muscle, and *C. pilosula* increased the average amplitude of contraction waves of longitudinal and circular muscles from gastric antrum.

Apart from the efficacy of HMs on alleviating GI toxicity, investigation of the safe use of HMs was another significant focus. Potential adverse effects of the combination of HMs with chemotherapy may appear due to direct toxic effects of HMs, herb–herb, or herb–drug interactions, which are considered major concerns among patients with CRC, especially for those undergoing active chemotherapy (56). However, commonly used HMs such as *C. reticulata, A. membranaceus* and SJZD are generally perceived as relatively safe treatments with rarely reported adverse effects (57–60). Studies on animal and clinical trials have shown the safety of these HMs. For example, a clinical and preclinical systematic review was conducted by Zheng et al. to investigate the safety of *A. membranaceus*. Twenty-eight RCTs with 2,522 participants and 16 animal studies with 634 animals were accessed (61). They concluded that *A. membranaceus* was a relatively safe herb as no statistical difference was found in the incidence of adverse reaction. Ma Jin-Yeul conducted a study on rats to determine the potential toxic effects of SJZD, and the results showed that no direct toxic effects or negative herb–herb interaction among *C. pilosula, G. uralensise, A. macrocephala*, and *Poria cocos (Schw.) Wolf*. were found (62). Nevertheless, most of the herbs commonly combined with chemotherapy have not been well-studied, and clinically relevant data on herb–drug interaction is sparse (63).

In our meta-analysis, although none of the included studies reported adverse effects related to HMs, only seven studies (21, 27, 30, 35–37, 39) stated that no adverse effects were caused during the trials; however, evidence was insufficient. Furthermore, RCTs may not reliably explore rare adverse effects or adverse effects with significant latency because of the limited sample size and time (64). Thus, a clear conclusion regarding the safety of HMs requires further investigation. Moreover, nine studies (21–25, 30, 35, 37, 39) showed that HMs intervention alleviated CIGI toxicity without causing a reduction in the response to chemotherapy. Among them, three studies (22, 23, 35) concluded that HMs had statistically significant anti-tumor effects in increasing tumor response. The HMs evaluated in these studies deserve to be further investigated by RCTs.

## Limitations

The following limitations of our meta-analysis are present. First, most of the included studies in our meta-analysis were performed in Chinese populations. Further investigation should be done to assess the efficacy of HMs on CIGI toxicity in other populations. Moreover, our meta-analysis did not include any unpublished study, although an attempt was made to retrieve it. Second, the same studies were used in duplicate in the analysis of the overall effect of HM on CIGI toxicity. This resulted in inflating the total sample size and overestimated the efficacy of HMs on alleviating overall CIGI toxicity. More well-designed RCTs should be conducted to support our conclusion. Third, the RoB occurred in many of the included studies, which limited the credibility of the results. More specifically, the lack of blinding in the control groups was the most significant bias in our meta-analysis. It likely produced a placebo effect, especially for less objective outcomes such as nausea, anorexia, and abdominal distension. Other sources of bias, such as low compliance with protocols and unclarity of HM ingredients in studies, and selective reporting of non-significant outcomes, may have influenced the reliability of the effect sizes. Moreover, most of the studies were carried out in single hospitals. Fourth, the heterogeneity of nausea and vomiting, anorexia, and diarrhea were observed to be medium. Heterogeneity was not eliminated by sensitivity analysis. Various toxicity criteria were used for CIGI toxicity evaluation, contributing to the heterogeneity of this meta-analysis. Differences in sample size, tumor stage and grade, and ingredients and doses of HMs were other possible factors causing heterogeneity.

## Conclusions

Although our meta-analysis showed that HMs intervention significantly alleviated overall CIGI toxicity, nausea and vomiting, diarrhea, oral mucositis, and abdominal distension without differentiation of methodological quality, sensitivity analysis of methodological quality revealed that a statistically significant effect of HMs is only shown in studies without a double-blind design. Therefore, further well-designed, double-blinded, large-scaled RCTs are warranted to comprehensively evaluate the treatment efficacy. Based on the ingredients of the HMs, further sensitivity analyses identified five herbs that showed consistent effects on alleviating CIGI toxicity in multiple combinations and multiple studies. However, at present, there are insufficient clinical trials of single herbs investigating the efficacy on treating CIGI toxicity in patients with CRC. Further clinical research includes the five herbs to chemotherapy in patients, the safety of the combinations of these herbs, and the potential synergistic effects of these combinations of herbs should be conducted.

## Data Availability Statement

The original contributions presented in the study are included in the article/Supplementary Material, further inquiries can be directed to the corresponding author/s.

## Author Contributions

YF conceived and designed the study. YC and C-sC developed the search terms and drafted the manuscript. H-YT, CT, and NW reviewed the protocol and revised the manuscript. All authors read and approved the final version of the manuscript.

## Conflict of Interest

The authors declare that the research was conducted in the absence of any commercial or financial relationships that could be construed as a potential conflict of interest.
