# Supplementary material for: Efficacy of Herbal Medicines Intervention for Colorectal Cancer Patients With Chemotherapy-Induced Gastrointestinal Toxicity — a Systematic Review and Meta-Analysis
Source: Front Oncol. 2021 Mar 25;11:629132. doi: 10.3389/fonc.2021.629132 (PMC8044744; doi:10.3389/fonc.2021.629132)
Supplement: Supplementary file 1 [file Table_1.DOCX]

Search strategy

PubMed

| 1 | unintended[tiab] OR undesirable[tiab] OR “side effect”[tiab] OR “adverse effect” [tiab] | 429,095 |
| --- | --- | --- |
| 2 | “chemotherapy-induced” [tiab] | 11,090 |
| 3 | "diarrhea"[MH] OR "constipation"[MH] OR "nausea"[MH] OR "vomiting"[MH] OR "anorexia"[MH] OR "abdominal pain"[MH] OR "dyspepsia"[MH] | 137,599 |
| 4 | chemotherapy[MH] OR chemotherap*[tiab] | 1,587,929 |
| 5 | (rectal[tiab] OR rectum[tiab] OR colon[tiab] OR colorectal[tiab] OR CRC[tiab]) AND (cancer*[tiab] OR neopla*[tiab] OR carcin*[tiab] OR tumor*[tiab] OR tumour*[tiab] OR malignan*[tiab]) | 251,233 |
| 6 | “plant extract”[tiab] OR “Chinese herbal”[tiab] OR “Chinese medicine”[tiab] OR herb*[tiab] OR "traditional medicine"[tiab] OR "alternative medicine"[tiab] OR "complementary medicine"[tiab] | 148,130 |
| 7 | “randomized controlled trial”[tiab] OR “controlled clinical trial”[tiab] OR ramdomized[tiab] OR ramdomised[tiab] OR placebo[tiab] OR sham[tiab] OR randomly[tiab] OR trial[tiab] | 1,054,605 |
| 8 | (#1 OR #2 OR #3) AND #4 AND #5 AND #6 AND #7 | 16 |

Web of Science

| 1 | TS=(unintended OR undesirable OR  “side effect” OR “side effects” OR “adverse effect” OR “adverse effects”) | 433,639 |
| --- | --- | --- |
| 2 | TS=(“chemotherapy-induced” ) | 14,133 |
| 3 | S=(("diarrhea") OR ("constipation") OR ("nausea") OR ("vomiting") OR ("anorexia") OR ("abdominal pain") OR ("dyspepsia") ) | 243,797 |
| 4 | TS= (chemotherap*) | 488,050 |
| 5 | TS=((rectal OR rectum OR colon OR colorectal) NEAR/10 (cancer* OR neopla* OR carcin* OR tumor* OR tumour* OR malignan*) ) | 310,253 |
| 6 | TS=(“plant extract” OR “Chinese herbal” OR “Chinese medicine” OR herb* OR "traditional medicine" OR "alternative medicine" OR "complementary medicine") | 300,516 |
| 7 | TS=(“randomized controlled trial” OR “controlled clinical trial” OR ramdomized OR ramdomised OR placebo OR sham OR randomly OR trial) | 2,041,843 |
| 8 | (#1 OR #2 OR #3) AND #4 AND #5 AND #6 AND #7 | 52 |

Cochrane

| 1 | (unintended OR undesirable OR “side effect” OR “side effects” OR “adverse effect” OR “adverse effects”) :ti,ab,kw | 264591 |
| --- | --- | --- |
| 2 | (chemotherapy-induced) :ti,ab,kw | 3845 |
| 3 | (("diarrhea") OR ("constipation") OR ("nausea") OR ("vomiting") OR ("anorexia") OR ("abdominal pain") OR ("dyspepsia")) :ti,ab,kw | 85142 |
| 4 | (chemotherap*) :ti,ab,kw | 80193 |
| 5 | (("rectal" OR "rectum" OR "colon" OR "colorectal") NEAR/10 (cancer* OR neopla* OR carcin* OR tumor* OR tumour* OR malignan*)) :ti,ab,kw | 22154 |
| 6 | (“plant extract” OR “Chinese herbal” OR “Chinese medicine” OR herb* OR "traditional medicine" OR "alternative medicine" OR "complementary medicine") :ti,ab,kw | 35253 |
| 7 | (“randomized controlled trial” OR “controlled clinical trial” OR ramdomized OR ramdomised OR placebo OR sham OR randomly OR trial) :ti,ab,kw | 1302398 |
| 8 | (#1 OR #2 OR #3) AND #4 AND #5 AND #6 AND #7 | 102 |
|  | in Trials | 69 |

Ovid MEDLINE

| 1 | (unintended OR undesirable OR “side effect” OR “side effects” OR “adverse effect” OR “adverse effects”) : ab | 55,058 |
| --- | --- | --- |
| 2 | (chemotherapy-induced) : ti,ab,kw | 1,419 |
| 3 | (("diarrhea") OR ("constipation") OR ("nausea") OR ("vomiting") OR ("anorexia") OR ("abdominal pain") OR ("dyspepsia")) :ti,ab,kw | 28,265 |
| 4 | (chemotherap*) :ti,ab,kw | 1,748 |
| 5 | (("rectal" OR "rectum" OR "colon" OR "colorectal") AND (cancer* OR neopla* OR carcin* OR tumor* OR tumour* OR malignan*)) :ab | 29,620 |
| 6 | (“plant extract” OR “Chinese herbal” OR “Chinese medicine” OR herb* OR "traditional medicine" OR "alternative medicine" OR "complementary medicine") :ab | 23,044 |
| 7 | (“randomized controlled trial” OR “controlled clinical trial” OR ramdomized OR ramdomised OR placebo OR sham OR randomly OR trial) :ti,ab,kw | 218,494 |
| 8 | (#1 OR #2 OR #3) AND #4 AND #5 AND #6 AND #7 | 2 |
|  | in Trials | 0 |

Embase

| 1 | ("unintended" or "undesirable" or "side effect" or "side effects" or "adverse effect" or "adverse effects").mp. [mp=title, abstract, heading word, drug trade name, original title, device manufacturer, drug manufacturer, device trade name, keyword, floating subheading word, candidate term word] | 1467237 |
| --- | --- | --- |
| 2 | chemotherapy-induced.mp. | 24917 |
| 3 | ("diarrhea" or "constipation" or "nausea" or "vomiting" or "anorexia" or "abdominal pain" or "dyspepsia").mp. [mp=title, abstract, heading word, drug trade name, original title, device manufacturer, drug manufacturer, device trade name, keyword, floating subheading word, candidate term word] | 793501 |
| 4 | chemotherap*.mp. | 878719 |
| 5 | (("rectal" or "rectum" or "colon" or "colorectal") and (cancer* or neopla* or carcin* or tumor* or tumour* or malignan*)).mp. [mp=title, abstract, heading word, drug trade name, original title, device manufacturer, drug manufacturer, device trade name, keyword, floating subheading word, candidate term word] | 498226 |
| 6 | ("plant extract" or "Chinese herbal" or "Chinese medicine" or herb* or "traditional medicine" or "alternative medicine" or "complementary medicine").mp. [mp=title, abstract, heading word, drug trade name, original title, device manufacturer, drug manufacturer, device trade name, keyword, floating subheading word, candidate term word] | 408737 |
| 7 | ("randomized controlled trial" or "controlled clinical trial" or ramdomized or ramdomised or placebo or sham or randomly or trial).mp. [mp=title, abstract, heading word, drug trade name, original title, device manufacturer, drug manufacturer, device trade name, keyword, floating subheading word, candidate term word] | 2664168 |
| 8 | (#1 OR #2 OR #3) AND #4 AND #5 AND #6 AND #7 | 169 |

CINAHL PLUS

| 1 | (unintended OR undesirable OR “side effect” OR “side effects” OR “adverse effect” OR “adverse effects”) : ab | 17,683 |
| --- | --- | --- |
| 2 | (chemotherapy-induced) : ti,ab,kw | 675 |
| 3 | (("diarrhea") OR ("constipation") OR ("nausea") OR ("vomiting") OR ("anorexia") OR ("abdominal pain") OR ("dyspepsia")) :ti,ab,kw | 9,987 |
| 4 | (chemotherap*) :ab | 12,626 |
| 5 | (("rectal" OR "rectum" OR "colon" OR "colorectal") AND (cancer* OR neopla* OR carcin* OR tumor* OR tumour* OR malignan*)) :ab | 7,250 |
| 6 | (“plant extract” OR “Chinese herbal” OR “Chinese medicine” OR herb* OR "traditional medicine" OR "alternative medicine" OR "complementary medicine") : ti,ab,kw | 15,190 |
| 7 | (“randomized controlled trial” OR “controlled clinical trial” OR ramdomized OR ramdomised OR placebo OR sham OR randomly OR trial) :ti,ab,kw | 106,247 |
| 8 | (#1 OR #2 OR #3) AND #4 AND #5 AND #6 AND #7 | 6 |

AMED

| 1 | (unintended OR undesirable OR “side effect” OR “side effects” OR “adverse effect” OR “adverse effects”) : ti,ab,kw | 9284 |
| --- | --- | --- |
| 2 | (chemotherapy-induced) : ti,ab,kw | 154 |
| 3 | (("diarrhea") OR ("constipation") OR ("nausea") OR ("vomiting") OR ("anorexia") OR ("abdominal pain") OR ("dyspepsia")) :ti,ab,kw | 2665 |
| 4 | (chemotherap*) : ti,ab,kw | 1555 |
| 5 | (("rectal" OR "rectum" OR "colon" OR "colorectal") AND (cancer* OR neopla* OR carcin* OR tumor* OR tumour* OR malignan*)) : ti,ab,kw | 1080 |
| 6 | ("plant extract" OR "Chinese herbal" OR "Chinese medicine" OR herb* OR "traditional medicine" OR "alternative medicine" OR "complementary medicine") : ti,ab,kw | 26991 |
| 7 | ("randomized controlled trial" OR "controlled clinical trial" OR ramdomized OR ramdomised OR placebo OR sham OR randomly OR trial) :ti,ab,kw | 18606 |
| 8 | (#1 OR #2 OR #3) AND #4 AND #5 AND #6 AND #7 | 0 |

China National Knowledge Infrastructure (CNKI)

(((((摘要=中药) OR 摘要=中医药) AND (((((摘要="大肠癌") OR "结肠癌") OR "直肠癌") OR "结直肠癌") OR "结肠直肠癌")) AND (摘要=化疗)) AND (((不良反应) OR 副作用) OR 并发症)) AND (摘要=临床)

WanFang Data

(((((摘要=中药) OR 摘要=中医药) AND (((((摘要="大肠癌") OR "结肠癌") OR "直肠癌") OR "结直肠癌") OR "结肠直肠癌")) AND (摘要=化疗)) AND (((不良反应) OR 副作用) OR 并发症)) AND (摘要=临床) AND (((((摘要=腹泻) OR 便秘) OR 痢疾) OR 泄泻) OR 大便不通) OR ((((恶心) OR 反胃) OR 作呕) OR 呕吐) OR (((((厌食) OR 食欲不振) OR 食欲缺乏) OR 腹痛) OR 消化不良) OR (((((消化道出血) OR 胃肠出血) OR 胃溃疡) OR 消化道溃疡) OR 胃出血)
