# Supplementary material for: Efficacy of Herbal Medicines Intervention for Colorectal Cancer Patients With Chemotherapy-Induced Gastrointestinal Toxicity — a Systematic Review and Meta-Analysis
Source: Front Oncol. 2021 Mar 25;11:629132. doi: 10.3389/fonc.2021.629132 (PMC8044744; doi:10.3389/fonc.2021.629132)
Supplement: Supplementary file 2 [file Table_2.DOCX]

| First author (year) | Toxicity assessment criteria |
| --- | --- |
| Xu, H. X. (2011) | WHO Criteria |
| Liu, S. J. (2014) | NS |
| Ji, H. (2016) | NS |
| Li, X. Q. (2019) | NS |
| Hu, A. L. (2015) | WHO Criteria |
| Hu, H. L. (2016) | Recommendations of the ESICM Working Group on Abdominal Problems |
| Xing, J. (2016) | Guidelines for Clinical Research of New Chinese Medicines |
| Huang, S. M. (2016) | Guidelines for Clinical Research of New Chinese Medicines |
| Chen, J. X. (2012) | Guidelines for Clinical Research of New Chinese Medicines |
| Xiao, H. (2011) | WHO Criteria |
| Zhang, X. L. (2015) | WHO Criteria |
| Zhao, Y. F. (2015) | WHO Criteria |
| Nan, B. (2016) | Guidelines for Clinical Research of New Chinese Medicines |
| Song, J. Q. (2019) | NS |
| Zhang, C. (2015) | Guidelines for Clinical Research of New Chinese Medicines |
| Peng, G. Q. (2016) | WHO Criteria |
| Zeng, H. F. (2018) | NS |
| Matsuda, C. (2015) | WHO Criteria |
| Motoo, Y. (2020) | National Cancer Institute Common Terminology Criteria for Adverse Events |
| Kono, T. (2013) | NS |
| Liu, Y. (2013) | National Cancer Institute Common Toxicity Criteria |
| Oki, E. (2015) | National Cancer Institute Common Terminology Criteria for Adverse Events |

Toxicity assessment criteria

NS: not stated; WHO: World Health Organization; ESICM: European Society of Intensive Care Medicine
